# Easing Intermediates Search by Combining Spectroscopy and Multivariate Curve Reconstruction: [Cu^I^(6,6′-dimethyl-2,2′-bipyridyl)_2_]PF_6_ Oxidation as Case Study

**DOI:** 10.1021/acs.jpclett.4c03467

**Published:** 2025-02-06

**Authors:** Gabriele Deplano, Isabelle Gerz, Derya Demirbas, Barbara Centrella, Matteo Bonomo, Serena DeBeer, Silvia Bordiga, Matteo Signorile, Sergio A. V. Jannuzzi

**Affiliations:** a Department of Chemistry, NIS and INSTM Reference Centre, Università di Torino, Via P. Giuria 7, 10125 and Via G. Quarello 15/A, 10135 Torino, Italy; b Department of Inorganic Spectroscopy, Max Planck Institute for Chemical Energy Conversion, Stiftstraße 34−36, 45470 Mülheim an der Ruhr, Germany; c Department of Molecular Theory and Spectroscopy, Max-Planck-Institut für Kohlenforschung, Kaiser-Wilhelm-Platz 1, 45470 Mülheim an der Ruhr, Germany

## Abstract

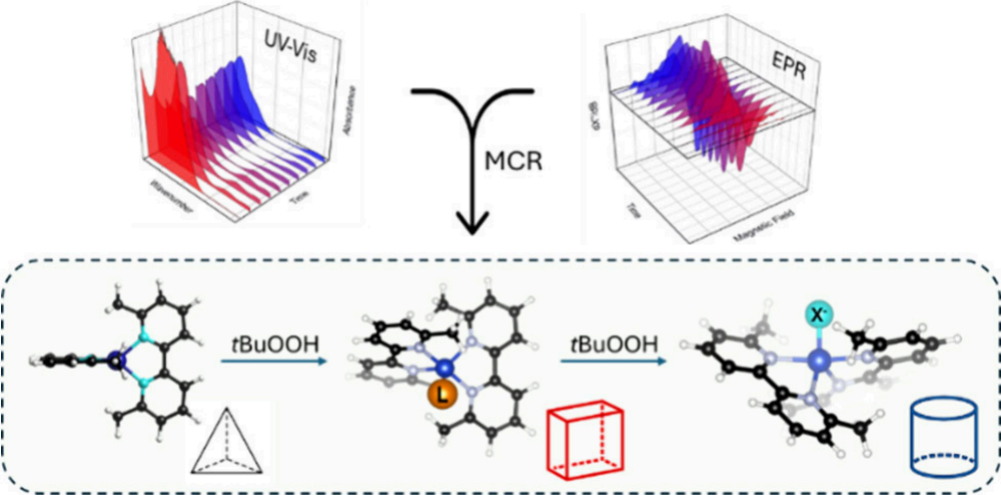

Despite their prevalence in catalysis, complex reaction mixtures are not trivial to investigate and disentangle. Different approaches can be applied to characterize them, even featuring low-dimensionality data sets. The liquid-phase reaction of [Cu^I^(6,6′-dimethyl-2,2′-bipyridyl)_2_]PF_6_ (**Cu**^**I**^) with *tert*-butyl hydroperoxide is investigated: two Cu^II^ species are found upon oxidation of the pristine complex, characterized by different spectroscopic and kinetics fingerprints. Coupling EPR and UV–vis spectroscopies with chemometric methods (namely, multivariate curve reconstruction, MCR) allowed for easily retrieving pure spectral features and concentration profiles. Spectrokinetic analysis independently showed an optimal agreement with kinetic outcomes from MCR. Finally, hypotheses on the nature of the Cu^II^ species are drawn on the basis of EPR fitting and quantum chemistry computations on a series of candidate structures. Beyond the accurate characterization of a model system, this study demonstrates the potential of coupling multivariate statistical techniques, experiments, and computations toward a quantitative understanding of electronic and kinetic information on complex chemical systems.

The oxidation–reduction dynamics of metal centers play a crucial role in various catalytic and biological systems. Although such types of systems are extensively studied, their complete understanding is often blurred by the complexity of reaction networks involved, yielding reaction mixtures comprising several intermediates with potentially similar chemical nature.^1−4^ As a model system, we investigate herein the oxidation of the [Cu^I^(6,6′-dimethyl-2,2′-bipyridyl)_2_]PF_6_ complex (**Cu**^**I**^) in solution in the presence of *tert*-butyl hydroperoxide (*t*BuOOH). **Cu**^**I**^ presented the best redox reversibility of its Cu metal center among other candidates, mainly because of the stabilizing effect of the 6,6′-dimethyl substitution on the ligand, and it has been recently reported as catalyst in the partial oxidation reaction of cyclohexene, producing 2-cyclohexen-1-ol and its overoxidized product 2-cyclohexen-1-one.^5^ This behavior is not surprising, as Cu complexes are renowned for their activity in partial oxidation reactions in the presence of *t*BuOOH.^6−20^ While a Cu-(hydro)peroxo intermediate is suggested for a tetranuclear complex with a tridentate ligand reacted with H_2_O_2_,^20−23^ the intermediates of 2,2′-bipyridine complexes in alkane and alkene oxidation reactions with *t*BuOOH remain unknown despite numerous reports.^18,24−28^

To probe the transient species and reaction intermediates involved in the oxidation of **Cu**^**I**^, this study employs an integrated approach that combines freeze-quench EPR and *in situ* UV–vis spectroscopies. These techniques provide invaluable real-time insights into the electronic states and coordination environments of the Cu species as the reaction progresses. However, the complexity of the spectral data arising from overlapping signals and the presence of multiple species in equilibrium makes a quantitative analysis (e.g., retrieval of spin-Hamiltonian parameters via EPR fitting) close to impossible without a reliable starting guess from the operator, potentially leading to a biased outcome. In this context, the use of multivariate statistical techniques can allow for a more robust interpretation of the experimental data, as single reaction components can be isolated and analyzed separately. Furthermore, by opportunely adopting multivariate curve resolution (MCR), we could simultaneously obtain reliable concentration profiles and pure spectral profiles of the individual species involved in the reaction. These profiles can then be rigorously analyzed and compared to reference/simulated spectra, allowing for the characterization of the transient species according to structural/electronic patterns.

Although multivariate techniques have seen widespread use in UV–vis spectroscopy for analyzing complex mixtures,^29−37^ their application to EPR spectroscopy, particularly in the context of metal complexes, remains relatively limited;^38−40^ this is particularly true for algorithms that go beyond principal component analysis and are able to extract significant kinetic data from data sets. The current study addresses this gap by demonstrating the utility of these techniques in conjunction with magnetic resonance and UV–vis spectroscopy. The results offer a detailed case study that not only advances our understanding of Cu-mediated oxidation processes but also showcases the potential of combining spectroscopic and statistical methodologies for the comprehensive analysis of complex chemical reactions involving copper centers.

The X-band EPR and UV–vis spectra of a 1:5 (mol:mol) solution of **Cu**^**I**^ and *t*BuOOH were collected at selected times during the reaction. The obtained spectral profiles are listed in Figure 1.

**Figure 1:**
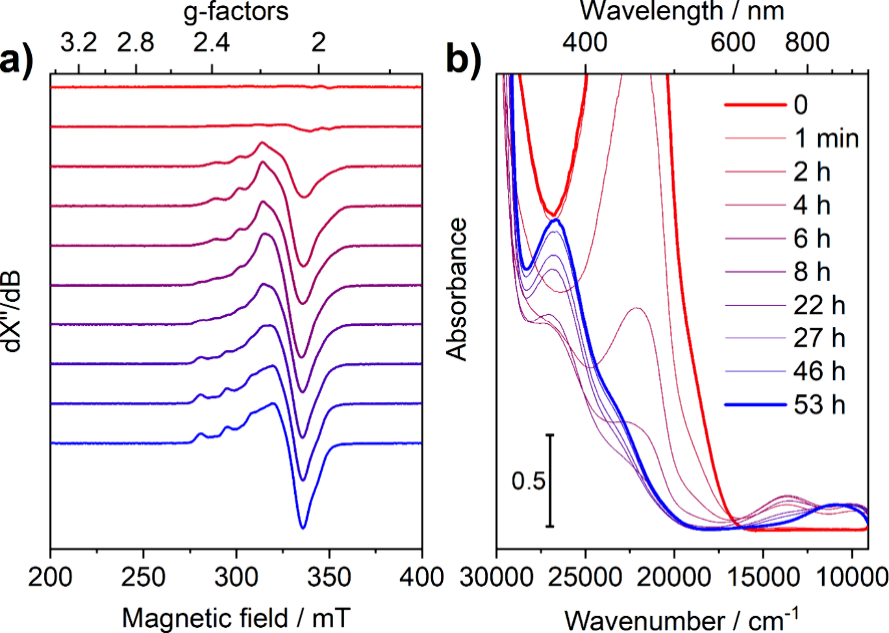
(a) X-band EPR measured at 30 K and (b) UV–vis (optical path 1.0 cm) spectra of 1 mM **Cu**^**I**^ in a 1:1 CH_2_Cl_2_/CH_3_CN mixture during reaction with *t*BuOOH (1:5 molar ratio). The legend reported in panel b refers to both panels.

The absence of any appreciable signal in the EPR spectrum of the starting solution (red spectrum in Figure 1a) is consistent with the presence of an EPR-silent d^10^ state of **Cu**^**I**^ at the start of the reaction, as further corroborated by the absence of d–d transitions in the 9000–17000 cm^–1^ region of the UV–vis spectrum. In contrast, the final state after 53 h of reaction (blue spectra in Figure 1) is assigned to a Cu^II^ species, both from the characteristic ^63,65^Cu (*I* = 3/2) EPR signal and from the presence of a broad, likely multicomponent d–d band at ∼11000 cm^–1^ in the UV–vis spectrum. Looking at intermediate states, it is clear that the conversion of **Cu**^**I**^ to the final state proceeds quantitatively via an intermediate Cu^II^ species (named **Cu**^**II**^**_1**) that is different from the final one (referred as **Cu**^**II**^**_2**). This is apparent by (a) the formation of a species with an EPR spectrum that is clearly different from the final one and (b) the transient appearance of two d–d bands centered at around 13 700 and 9700 cm^–1^ in the UV–vis spectrum.

Hyperfine coupling constants and *g*-values extracted from EPR spectra can supply useful geometric/electronic information about the species responsible for the EPR signal; although software like EasySpin^41^ are routinely used to fit EPR spectra to retrieve these parameters, high-dimensionality data sets can severely hinder the fitting ability of such software in terms of computational cost and reliability given the multitude of local minima. In the present case, supposing two EPR-active species are formed during reaction, 3 elements of the **g**- and **A**-matrices need to be refined for each species to fit the experimental ensemble of spectra, together with the relative proportion of the two species for each spectrum at each time point. Additionally, a line width parameter (H strain) needs to be fitted for the three axes of each species. Isotropic broadening fails to describe the particular low-field strain on these spectra, as already well documented in the case of Cu^II^ compounds.^42^ Considering all 10 time points, a total of 28 parameters make a global fit impractical. Reliable results for the spin-Hamiltonian parameters could, in principle, come by fitting the spectrum referring to a mixed time point, although 19 parameters are involved. Indeed, we attempted fitting the mixture spectrum at 22 h of reaction, when a comparable concentration of **Cu**^**II**^**_1** and **Cu**^**II**^**_2** is expected. A standard fit with a reasonable initial guess failed to converge with the standard Nelder–Mead simplex algorithm over 6 h on a personal computer. Accordingly, to perform a fit with minimal bias from the operator in a reasonable time frame (typically a few h/fit), we propose a dedicated Monte Carlo algorithm requiring as input only the maximal ranges within the random generation of parameters that is performed. As an additional feature, these ranges are modified after each *n* iterations, by restricting them proportionally to the fit RMSD (see Figure S1). Full details on this adaptive Monte Carlo (AMC) optimizer are provided together with the full Matlab code in the Supporting Information. The fit has been repeated 10 times, each starting from different and randomly selected initial guess. By a visual inspection of fit results (Figure S2), most of the repetitions yield point-to-point agreement with the experimental data, with satisfactory RMSD values. However, there is no consistency among the spin-Hamiltonian and the compositional parameters obtained along the different repetitions (Table S1). For this reason, the EPR parameters for **Cu**^**II**^**_1** and **Cu**^**II**^**_2** and their relative abundance cannot be unambiguously derived through this direct approach.

Steinbock et al. proposed a possible solution to this problem by applying principal component analysis to retrieve the pure spectral profiles of compounds in a mixture.^39^ A similar approach could be exploited to significantly reduce the dimensionality of the problem by extracting pure spectral components from the data set (while robustly evaluating the number of species) and obtaining spin-Hamiltonian parameters from independent fits of the pure spectra. Multivariate methods have been widely used in the past years to aid the interpretation of spectral profiles that show some significant variance along a selected experimental parameter (e.g., time, temperature, concentration of a reagent).^43^ In particular, the ability of the MCR (here implemented with the alternate least-squares, ALS, algorithm) to retrieve pure spectral and concentration profiles from a complex data set has been exploited to study systems in which the concentration of a specific species is never present as the sole component of the mixture, and no reference compound for such species is known *a priori*. This algorithm was thus simultaneously applied to the EPR and UV–vis spectra presented in Figure 1, providing a set of pure-component spectra best fitting both spectroscopic data sets simultaneously, as reported in Figure 2.

**Figure 2:**
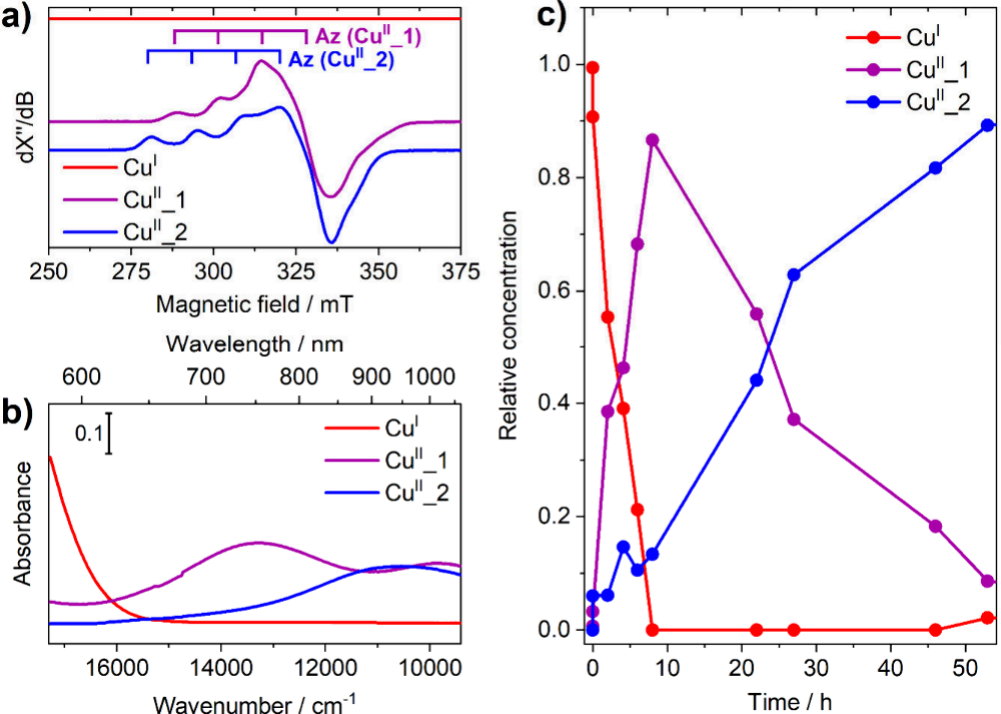
Pure spectral profiles obtained from MCR-ALS for (a) EPR and (b) UV–vis. (c) Pure concentration profiles obtained from MCR-ALS. *R*^2^ = 0.984, lack of fit = 12.8%. The kink at ∼15 000 cm^–1^ in the UV–Vis spectrum is an artifact due to a detector switch in the instrument.

The UV–vis component corresponding to the initial **Cu**^**I**^ state consists of the tail of the MLCT band at 21 636 cm^–1^,^44^ whereas the corresponding EPR profile has negligible intensity as expected. The specific differences between components associated with **Cu**^**II**^**_1** and **Cu**^**II**^**_2** are evident in the pure spectral profiles: their pure EPR spectra (Figure 2a) present significant strain in the parallel component, especially **Cu**^**II**^**_1**, making it consistent with a more rhombic **g**-tensor compared to the more axial one for **Cu**^**II**^**_2**. Both pure EPR spectra show the resolved hyperfine lines, as expected from the type II site of the ^63,65^Cu (*I* = 3/2) nucleus, demonstrating that the MCR reconstruction yielded physically sound results. If 5-coordinated species are hypothesized to form, these would be consistent with geometries closer to trigonal bipyramidal and square-based pyramidal, respectively. The spectrum of **Cu**^**II**^**_1** further resembles that of rhombic reference compounds [Cu^II^(6,6′-dimethyl-2,2′-bipyridyl)_2_(H_2_O)](OTf)_2_ (**R1**) and [Cu^II^(6,6′-dimethyl-2,2′-bipyridyl)_2_(CH_3_CN)](ClO_4_)_2_ (**R2**) in the same solvent (Figure S3). On the UV–vis side (Figure 2b), the profile of **Cu**^**II**^**_1** in the d–d spectral region corresponds to that which we have previously reported,^44^ presenting the characteristic transitions at 13 701 and 9671 cm^–1^. The spectrum of **Cu**^**II**^**_2**, instead, reveals a single broad/asymmetric band at 10 914 cm^–1^, possibly composed of two overlapping contributions. The concentration profiles (Figure 2c) confirm that the conversion of **Cu**^**I**^ → **Cu**^**II**^**_1** is faster than **Cu**^**II**^**_1** → **Cu**^**II**^**_2**: in fact, **Cu**^**II**^**_1** reaches its maximum concentration around 8 h of reaction, while **Cu**^**II**^**_2** becomes the major component only after ∼30 h. A semiquantitative fit of the concentration profiles (supposing first-order kinetics for all species involved) leads to an estimate of *k*_1_ ≈ 5·*k*_2_ (see Figure S4 and description herein), with *k*_1_ and *k*_2_ corresponding to the rate constant for **Cu**^**I**^ → **Cu**^**II**^**_1** and **Cu**^**II**^**_1** → **Cu**^**II**^**_2**, respectively.

To further validate the approach, we independently modeled the kinetics of the processes through a dedicated UV–vis spectrokinetic study performed varying the initial **Cu**^**I**^:*t*BuOOH molar ratio (ranging from 1:1 to 1:200; full details are provided in the Supporting Information, Figures S5 and S6 and Table S2). *k*_1_ and *k*_2_ were determined to be (1.4 ± 0.1) × 10^–3^ s^–1^·M^–0.5^ and (4.0 ± 0.4) × 10^–4^ s^–1^·M^–0.67^, respectively (i.e., *k*_1_ = 3.5·*k*_2_; see Table S3). Given these values and integrating the rate equations, the concentration profiles for **Cu**^**I**^, **Cu**^**II**^**_1** and **Cu**^**II**^**_2** can be retrieved and compared to the analogous MCR concentrations (Figure 3).

**Figure 3:**
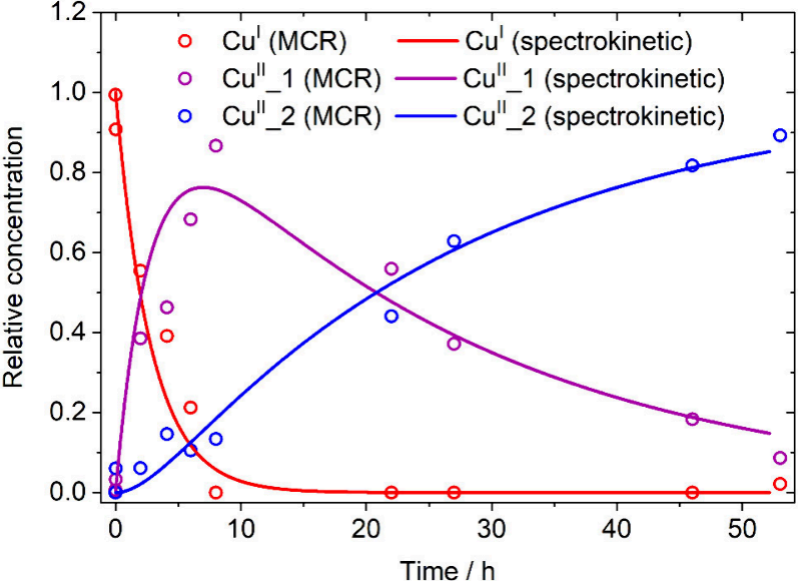
Concentration profiles obtained from the UV–vis spectrokinetic model (full lines, considering a **Cu**^**I**^:*t*BuOOH molar ratio 1:5), compared with those from MCR-ALS reconstruction (empty dots).

The agreement of the spectrokinetic concentration profiles with MCR-ALS data is excellent (*R*^2^ = 0.969), also considering the low dimensionality of the data set adopted in the reconstruction. Furthermore, the relative amounts obtained by MCR are in line with the spin quantification curve (Figure S5), which is, in turn, consistent with the total amount of Cu within the expected experimental error. These findings underscore the reliability of the MCR-ALS decomposition in this context. Additionally, the spectrokinetic study allowed the reaction order determination, which has been found equal to 1 for both **Cu**^**I**^ in the first reaction and **Cu**^**II**^**_1** in the second one, whereas it has been shown to be fractional in both reaction steps for *t*BuOOH (0.5 in first reaction, 0.67 for the second one). A fractional reaction order suggests that the two processes are not elementary reaction steps. While dedicated studies with tailored approaches to probe short-lived intermediates fall beyond the scope of this work, the reaction between **Cu**^**I**^ and *t*BuOOH demonstrates the applicability of the MRC-ALS methodology in reactions with complex rate laws.

Having assessed the reliability of the MCR-ALS outcomes, the resulting pure spectra can be further analyzed to provide direct structural insights into the nature of the unknown **Cu**^**II**^**_1** and **Cu**^**II**^**_2** species. In this regard, EPR fitting constitutes a very powerful tool, the application of which is now much more straightforward than that on the experimental spectra (i.e., mixtures of **Cu**^**II**^**_1** and **Cu**^**II**^**_2**). The pure EPR spectra were fitted with the AMC solver, repeating the procedure 10 times per species starting from different random initial guesses. In this way, a best fit set of spin-Hamiltonian parameters can be obtained by averaging the results from single repetitions (preliminary discarding outliers), further offering an estimate on their error bar via standard deviation. The best fits for **Cu**^**II**^**_1** and **Cu**^**II**^**_2** are shown in Figure 4 (single fits and related spin-Hamiltonian parameters in Figures S8 and S9 and Tables S4 and S5). The fit results confirm the aforementioned strain on the parallel component for both species, particularly marked in the case of **Cu**^**II**^**_1**. It is also clear that despite the good quality of both fits, the derived spin-Hamiltonian parameters are poorly reliable with respect to their *x* and *y* components, in particular concerning the hyperfine tensor (determined with a relative error up to 100%). The *g*_*z*_ and *A*_*z*_ components are instead determined with much higher accuracy (relative error of <5%) and can therefore be reliably exploited as structural descriptor of the two species. In an attempt to pinpoint their exact chemical nature, an extensive DFT study on possible pentacoordinated [Cu^II^(6,6′-dimethyl-2,2′-bipyridyl)_2_(X)^*n*−^]^(2–*n*)+^ adducts (being X an extra ligand, either neutral or charged; see Figure S10) was performed. The *g*_*z*_ and *A*_*z*_ values from DFT have then been compared with the fit results, as depicted in Figure 4c.

**Figure 4:**
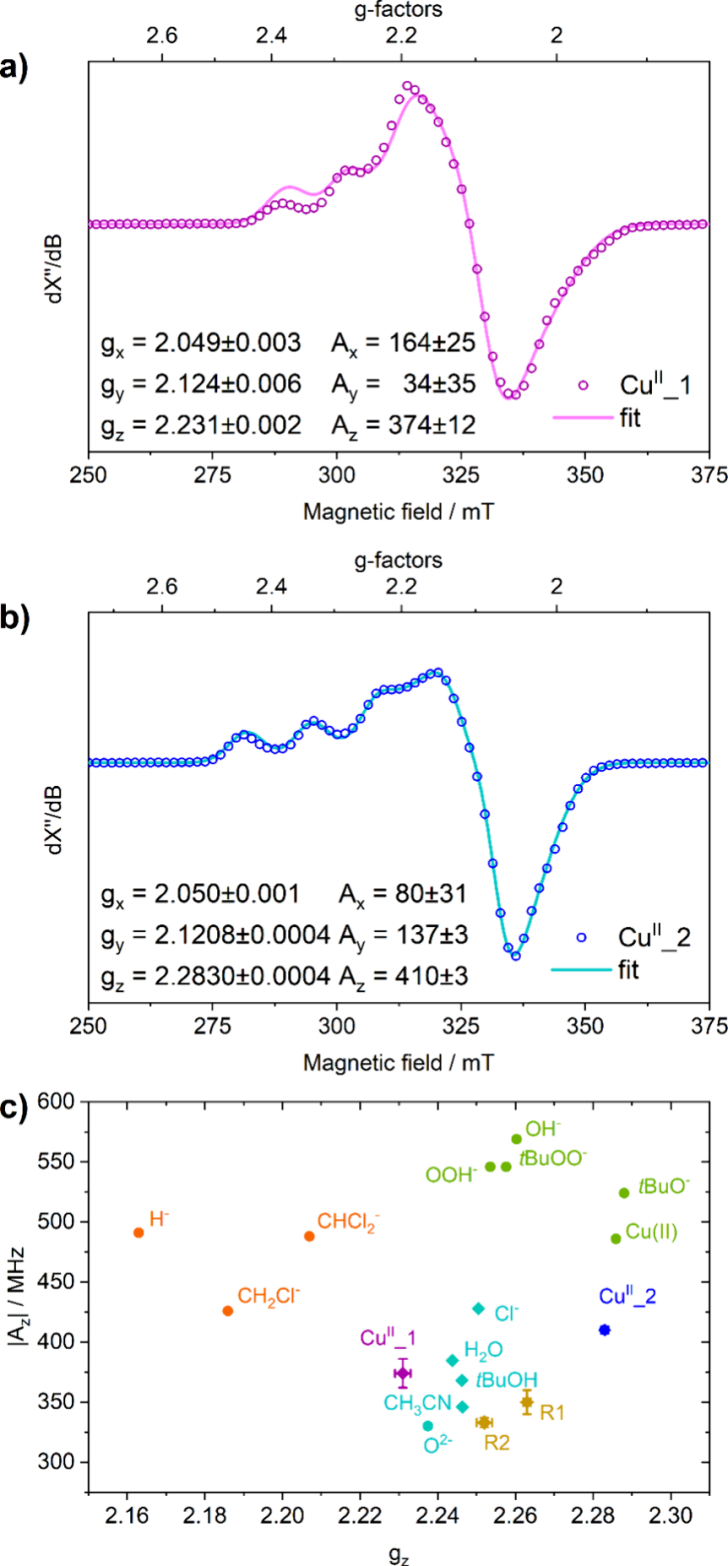
Experimental (empty dots) vs fitted (full lines) EPR spectra and main parameters for (a) **Cu**^**II**^**_1** and (b) **Cu**^**II**^**_2** (A in MHz); the best fits for **Cu**^**II**^**_1** and **Cu**^**II**^**_2** were simulated from averaged EPR parameters obtained from the sets portrayed in Figures S8 and S9 (excluding outliers, see section S1 of Supporting Information for full details), respectively. In the experimental spectra, only 1 point in every 8 is shown for the sake of visualization. (c) Fitted spin-Hamintonian parameters **Cu**^**II**^**_1** and **Cu**^**II**^**_2** compared to those computed for pentacoordinate [Cu^II^(6,6′-dimethyl-2,2′-bipyridyl)_2_(X)^*n*−^]^(2–*n*)+^ adducts; the point labeled as “Cu(II)” is relative to the calculated [Cu^II^(6,6′-dimethyl-2,2′-bipyridyl)_2_]^2+^ complex without additional ligands. **R1** (X = H_2_O) and **R2** (X = CH_3_CN) experimental references are included too. Axial and rhombic **g**-tensors are represented by circles and diamonds respectively (**g**-tensors were considered rhombic if |*g*_*x*_ – *g*_*y*_| > 0.07). The coloring of symbols for DFT models groups different classes of species depending on their spin-Hamiltonians parameters (based on a spectral clustering algorithm; see text for details).

The DFT calculated values are consistent with those obtained on the benchmark set of Cu-based complexes used to develop the computational method applied herein;^45,46^ this is particularly significant considering that no pentacoordinated 4N + 1X (X = O, N, Cl, ...) ligation pattern (i.e., the one proposed for both **Cu**^**II**^**_1** and **Cu**^**II**^**_2** and calculated for all candidate adducts) was present in the original data set. It must be stated, however, that most of the experimental data used in the study were collected in aqueous solution, with consequent possible effects on the spin-Hamiltonian parameters; direct comparison with the data in the present study, collected on apolar organic solvents, should be done with due care. A cluster analysis subdivides the computational results into three classes of species according to spin-Hamiltonian values: (i) adducts with monovalent anions binding Cu via an O atom and [Cu^II^(6,6′-dimethyl-2,2′-bipyridyl)_2_]^2+^ (green scatters), characterized by axial **g**-tensors, high *g*_*z*_, and high |*A*_*z*_| values; (ii) adducts with mainly neutral ligands (cyan scatters), characterized by rhombic **g**-tensors, medium-high *g*_*z*_, and low |*A*_*z*_| values; and (iii) adducts with monovalent anions binding Cu via an H or C atom (orange scatters), characterized by axial **g**-tensors, low *g*_*z*_, and medium-high |*A*_*z*_| values.

Comparison between experimental and computational data, also considering the references **R1** and **R2**, suggests that **Cu**^**II**^**_1** should be an adduct with a small, neutral species or a Cl^–^ ion as extra ligand; although CH_2_Cl_2_ is usually regarded as a noncoordinating, inert solvent, reports of a less innocent role of this molecule exist, where Cl^–^ ions were generated in solution via radical pathways in the presence of Cu.^47−49^ The possibility of a chloride complex in this case is however unlikely, since the cyclovoltammetry data from our previous study concerning this species differ from the one previously reported for [Cu^II^(6,6′-dimethyl-2,2′-bipyridyl)_2_(Cl)]Cl.^50^ In contrast, although the values fitted for **Cu**^**II**^**_2** are not close to the clusters defined by the models considered herein, this species should comprise an adduct with negatively charged O-containing species (e.g., OH^–^, OOH^–^, *t*BuO^–^, ...). The reason is its ability to transfer an oxygen atom to cyclohexene and restore the Cu(I) form.^5^ Inclusion of the PF_6_^–^ counterion and explicit solvent molecules using a multilevel optimization scheme (Figure S11) did not result in significant changes of the EPR parameters for the [Cu^II^(6,6′-dimethyl-2,2′-bipyridyl)_2_(OH)]PF_6_ model (Table S6), so they were excluded for all models; this negligible influence is consistent with the low coordinating ability of both PF_6_^–^ as a counterion and CH_2_Cl_2_ as a solvent.^51^ The possibility of the EPR spectra derived from MCR-ALS analysis comprising a mixture of chemical species^52^ cannot be ruled out.

In summary, the MCR-aided EPR and UV–vis study of the oxidation reaction of **Cu**^**I**^ by *t*BuOOH shows how tackling intermediates of a chemical reaction could require a powerful methodology to achieve a comprehensive description. The one-electron Cu^I^/Cu^II^ conversion coupled with the possibility of increased coordination number underscores how *in situ* multitechnique characterization can be necessary to disentangle the composition of reaction mixtures with peroxides despite the slow kinetics in the present case. Furthermore, the role of MCR algorithms to decompose complex data set comprised of mixtures of spectra was found to be fundamental to obtain fine details on the species that form, with the added benefit of retrieving concentration profiles of the system under investigation. Standard fitting procedures usually employed to extract spin-Hamiltonian parameters from EPR spectra proved to be particularly challenging in a direct application to raw experimental data in our case. Instead, decomposition of the initial data set using MCR provided a robust and easy tool to retrieve accurate information with the added value of concentration profiles. Although the assignment of the exact chemical identity of **Cu**^**II**^**_1** and **Cu**^**II**^**_2** remains ambiguous, many possible species were excluded, and general geometrical/electronic requirements for the remaining candidates have been established. All the presented results were based on the analysis of pure spectral profiles, showing the key role of MCR algorithms in facilitating the interpretation of *in situ* data sets from different spectroscopic techniques. While these methods have been successfully applied in other fields of spectroscopy to characterize metal-based systems, especially in the XAS field, the key information that can be extracted from even low-dimensionality spectroscopic data set using other techniques is still largely underutilized. Therefore, we postulate that the method disclosed herein is potentially applicable to any *in situ* multitechnique spectroscopic characterization of reaction mixtures.

## Experimental and Computational Methods

### Synthesis

 of **Cu**^**I**^,^53^**R1**,^54^ and **R2**(55) was performed according to literature (full procedures reported in the Supporting Information).

*UV–vis* spectra were collected on a dispersive Agilent 8454 spectrophotometer on a 1.0 cm cuvette with a 40 mL reservoir and a septum stopper. Sample aliquots were extracted with a micropipet after each UV–vis spectrum and frozen for EPR analysis.

*Continuous wave X-band EPR* spectra were collected on a Bruker E500 ELEXSYS spectrometer system equipped with an ER4116DM dual-mode cavity and an Oxford Instruments ESR 900 continuous-flow liquid helium cryostat interfaced with an ITC Mercury temperature controller (3.8–300 K range). The microwave unit was a high sensitivity ER049X Bruker superX bridge with an integrated microwave frequency counter. The magnetic field controller ER083CS was calibrated externally using an ER035M Bruker NMR field probe. The power used in all experiments was set to 0.19 mW to avoid saturation (Figure S12). Microwave frequency was 9.64 GHz, modulation frequency 100 kHz, modulation amplitude 0.75 mT, and temperature 30 K.

*EPR spectral simulations and fitting* were carried out with EasySpin 6.0.4 package^41^ running in parallelized Matlab via Parallel Toolbox with 4 parallel workers on a personal computer. The description of the adaptive Montecarlo algorithm and the script is given in the Supporting Information.

*MCR-ALS* was applied simultaneously on UV–vis and EPR spectra using the graphical user interface developed by Jaumot et al.^56^ Closure constraint was imposed on concentrations, whereas non-negativity was imposed for the UV–vis spectral data subset.

UV–vis spectra for the spectrokinetic analysis were collected on a Varian Cary5000 spectrophotometer with a screw-capped 1.0 cm quartz cuvette (QS grade) for the detection in the d–d transition zone (8000–16000 cm^–1^). An Avantes AvaSpec-ULS2048XL-EVO fiber optics spectrometer (25 μm slits, 100 μm core diameter high-OH fused silica fibers), coupled to an Avantes AvaLight-DH-S light source, was employed for the detection in the CT transitions zone (17000–28000 cm^–1^), using Hellma flow-through quartz glass (QS grade) cuvettes of 0.1 cm.

*DFT calculations* were performed with the ORCA 5.0.3 code.^57−59^ All structures were optimized using the hybrid B3LYP functional^60,61^ and the def2-TZVP basis set, including dispersive forces through the Grimme D3 empirical scheme with Becke–Johnson damping.^62^^63^ The effect of solvation was implicitly accounted for via the polarizable conductor calculation model (CPCM) method.^64−66^ EPR parameters were calculated on the resulting optimized structures: calculation of the **g**-tensor values were performed within the ZORA approximation^67^ using the double-hybrid PBE0-DH functional,^68^ ZORA-def2-TZVPP basis set for Cu, and the ZORA-def2-TZVP basis set for all other atoms.^69^ All other settings were chosen according to the detailed study by Drosou et al.^45^ Calculation of the **A**-tensor values were performed using hybrid B3PW91 functional,^60,70^ aug-cc-pVTZ-J basis set^71^ for Cu, and the ZORA-def2-TZVP basis set^69^ for all other atoms. All other settings were chosen according to the detailed study by Gómez-Piñeiro et al.^46^

Extended experimental and computational details are available in the Supporting Information.

## Data Availability

All data generated and analyzed in this study are available in the Edmond Open Research Data Repository at https://doi.org/10.17617/3.OWKBPS.

## Supporting Information Available

Description of AMC optimizer; fit of experimental EPR spectral mixture by AMC; EPR spectra of reference compounds; semiquantitative fit of MCR profiles; UV–vis spectrokinetic analysis; spin quantification; EPR fitting of Cu^II^_1 and Cu^II^_2; DFT structures; extended experimental and computational methods; Appendix I of AMC Matlab script; Appendix II of .xyz optimized DFT geometries; Appendix III of ORCA input examples (PDF)
