# Supplementary material for: Easing Intermediates Search by Combining Spectroscopy and Multivariate Curve Reconstruction: [CuI(6,6′-dimethyl-2,2′-bipyridyl)2]PF6 Oxidation as Case Study
Source: J Phys Chem Lett. 2025 Feb 6;16(7):1652–9. doi: 10.1021/acs.jpclett.4c03467 (PMC11849003; doi:10.1021/acs.jpclett.4c03467)
Supplement: Supplementary file 1 — jz4c03467_si_001.pdf [file jz4c03467_si_001.pdf]

Supporting Information for

Easing intermediates search by combining spectroscopy and multivariate curve reconstruction: $[Cu^{I}(6,6'\text{-dimethyl-}2,2'\text{-bipyridyl})_2]PF_6$ oxidation as case study

Gabriele Deplano[a], Isabelle Gerz[b], Derya Demirbas[c], Barbara Centrella[a], Matteo Bonomo[a], Serena DeBeer[b], Silvia Bordiga[a], Matteo Signorile[a] and Sergio A. V. Jannuzzi[b]**

[a] Department of Chemistry, NIS and INSTM Reference Centre, Università di Torino, Via P. Giuria 7, 10125 and Via G. Quarello 15/A, 10135, Torino, Italy

[b] Department of Inorganic Spectroscopy, Max Planck Institute for Chemical Energy Conversion, Stiftstraße 34–36, 45470 Mülheim an der Ruhr, Germany

[c] Department of Molecular Theory and Spectroscopy, Max-Planck-Institut für Kohlenforschung, Kaiser-Wilhelm-Platz 1, 45470 Mülheim an der Ruhr, Germany

Corresponding Author

Matteo Signorile, matteo.signorile@unito.it

Sergio A. V. Jannuzzi, sergio.jannuzzi@cec.mpg.de

S1. Adaptive Montecarlo optimizer

The adaptive Montecarlo (AMC) optimizer developed herein differs from the standard one implemented in Easyspin by the continuous adaptation of the allowed span range for parameters in a proportional way to the goodness of the fit (evaluated by RMSD). The workflow of the optimizer is shown schematically in Figure S1.

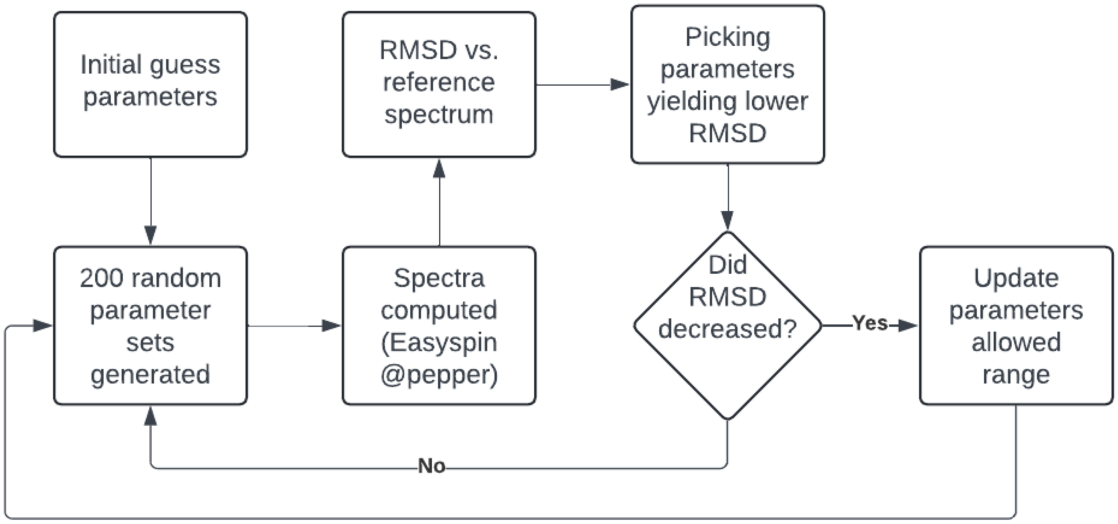

Figure S1. Flowchart of a search loop of the AMC optimizer. This is repeated until at least a convergence criterion is met.

An initial range for parameter generation is provided (*e.g.*, g=2.0-2.3, A=0-500 MHz, HStrain=1-500 MHz) and g/A tensor are initiated, then the search loop begins: *n* random sets of parameters (300 here) are generated, spectra are simulated with Easyspin (*pepper* function) and are compared with the reference spectrum (RMSD is computed after opportune normalization). The random search stage is parallelized (via Matlab® Parallel Toolbox) to speed-up the procedure (tested on both regular personal computer and cluster nodes with satisfactory scaling vs. the number of cores). As the *n* spectra and their RMSDs vs reference are computed, the set of

parameters providing the lower RMSD is selected. If its RMSD value is lower than that of previous cycle, the allowed range for random parameter generation is narrowed proportionally to the RMSD value. An "adaption coefficient" *ad* tunes the extent of the adjustment (set here to 0.02). The search loop is repeated until one among the following convergence criteria is satisfied:

- RMSD is lower than a given tolerance *tolfun*;
- RMSD variation is lower than *tolfun*/1000 for 3 consecutive cycles;
- RMSD is not decreasing for 10 consecutive cycles.

The full Matlab® scripts for AMC fit are provided in Appendix I.

The AMC fit procedure was repeated 10 times per spectrum, providing the possibility to statistically analyze the fit results. The results were filtered removing those fits deviating significantly from the average trend. A fit was discarded if at least 3 fit parameters deviate from the average by more than a standard deviation. Upon filtering, average and standard deviation of fit parameters were calculated. Best fit spectra were simulated from these averaged EPR parameters. We want to underline the choice off the *n* and *ad* parameters can greatly impact on the quality of the fit, in particular considering the reproducibility of the result across different repetitions of the procedure. We propose here a setting (n = 300, ad = 0.02) that allows for reasonable convergence in relatively short execution time. The probability of having a decrease in RMSD increases as *n* increases, as more trial sets of EPR parameters are generated. Please notice the execution time linearly scales wit *n*. Increasing *ad* will instead enhance the capability of better exploring the parameters space, as search rages will remain large enough to cross local maxima; the overall stability of the method could however decrease, leading to unpredictably increased execution time, or even to lack of convergence,

S2. Fit of experimental EPR spectral mixture by AMC

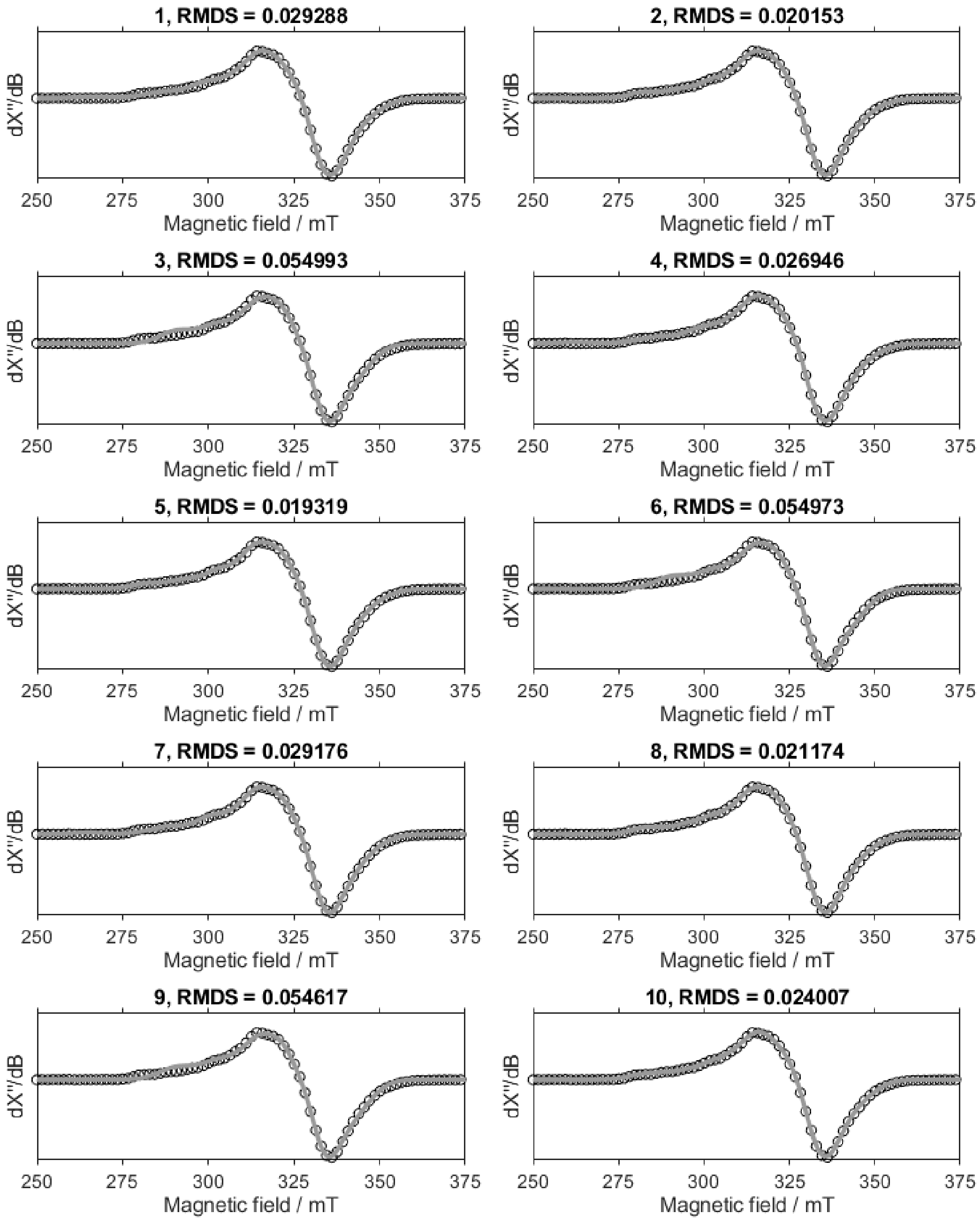

Figure S2. Experimental (empty dots) vs. fitted (full lines) EPR spectrum after 22 h of reaction. The fitting procedure has been repeated 10 times (AMC optimizer). In experimental spectra, only 1 point over 8 is shown for the sake of visualization. The full set of fitted parameters is provided in Table S1.

Table S1. Spin-Hamiltonian parameters obtained by 10 repeated fits (AMC optimizer) of the experimental mixture spectrum collected at 22 h. The values for A and H are expressed in MHz. The relative concentration of each component is given by the fractional parameter c, with $0 \leq c \leq 1$.

	**Component 1**										**Component 2**										
#	g_x	g_y	g_z	A_x	A_y	A_z	H_x	H_y	H_z	c	g_x	g_y	g_z	A_x	A_y	A_z	H_x	H_y	H_z	c	RMSD
1	2.0713	2.1174	2.2123	61	113	327	496	249	585	0.85	2.0106	2.2243	2.3245	104	421	633	263	231	406	0.15	0.02929
2	2.1636	2.2029	2.2533	379	332	481	355	307	298	0.18	2.0416	2.0986	2.2285	124	99	172	479	233	445	0.82	0.02015
3	2.0735	2.1014	2.2353	222	51	357	358	225	351	1.00	2.1850	2.2169	2.2926	310	294	418	72	102	190	0.00	0.05499
4	2.0724	2.1186	2.2122	33	110	334	503	253	609	0.85	2.0086	2.2208	2.3333	81	419	703	343	219	378	0.15	0.02695
5	2.1038	2.2079	2.2464	424	314	495	373	274	365	0.24	2.0424	2.1008	2.2261	68	105	149	454	216	400	0.76	0.01932
6	2.0745	2.1020	2.2340	228	44	363	340	199	367	1.00	2.1382	2.1544	2.2591	382	116	430	85	40	302	0.00	0.05497
7	2.0515	2.1249	2.2079	118	72	320	464	304	526	0.84	2.1025	2.2177	2.3246	31	383	675	188	216	374	0.16	0.02918
8	2.1108	2.2013	2.2436	450	313	487	360	306	311	0.24	2.0429	2.0995	2.2283	93	99	158	417	234	345	0.76	0.02117
9	2.0748	2.1022	2.2337	227	45	363	353	201	369	1.00	2.0998	2.2103	2.2605	174	295	418	187	163	265	0.00	0.05462
10	2.1156	2.1945	2.2462	110	319	471	367	356	402	0.35	2.0605	2.0983	2.1750	225	23	232	338	318	389	0.65	0.02401

S3. EPR spectra of reference compounds

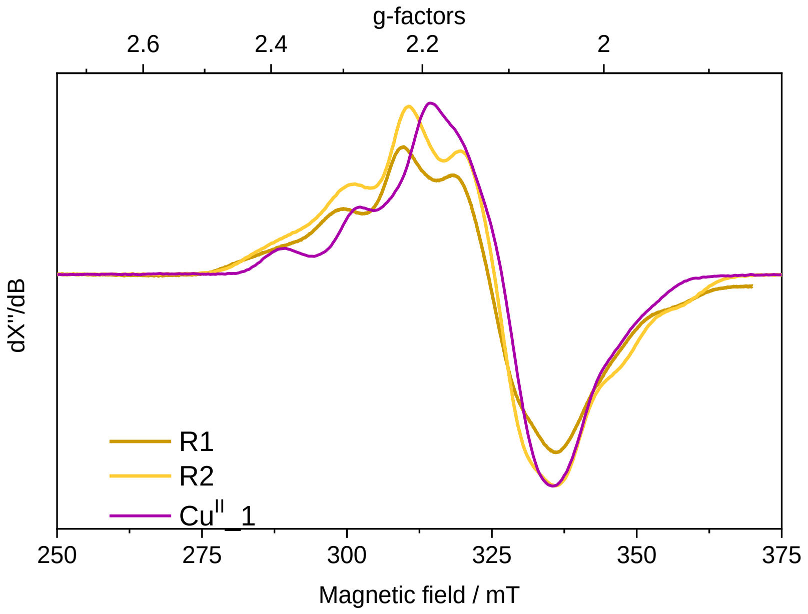

Figure S3. X-band EPR spectra of a 1 mM solution of **R1** ([Cu^{II}(6,6'-dimethyl-2,2'-bipyridyl)$_2$(H_2O)]($OTf)_2$) and **R2** ([Cu^{II}(6,6'-dimethyl-2,2'-bipyridyl)$_2$(CH_3CN)]($ClO_4)_2$).

S4. Semi-quantitative fit of MCR profiles

The concentration profiles obtained by MCR were initially fit considering a simple model consisting of consecutive reactions (Eq. S1):

$$\boldsymbol{Cu^{I} \overset{k_1}{\rightarrow} Cu^{II}\_1 \overset{k_2}{\rightarrow} Cu^{II}\_2} \tag{S1}$$

where **Cu^{I}** is the starting reagent, **Cu^{II}_1** is an intermediate and **Cu^{II}_2** is the final product; the only allowed (irreversible) reactions were set to be those from **Cu^{I}** to **Cu^{II}_1** and from **Cu^{II}_1** to **Cu^{II}_2**, which were modulated by the rate constants k_1 and k_2, respectively. The rate laws according to simple monomolecular kinetics for all species are shown in Eq. S2-S4:

$$\frac{d[Cu^I]}{dt} = -k_1[Cu^I] \tag{S2}$$

$$\frac{d[Cu^{II}\_1]}{dt} = k_1[Cu^I] - k_2[Cu^{II}\_1] \tag{S3}$$

$$\frac{d[Cu^{II}\_2]}{dt} = k_2[Cu^{II}\_1] \tag{S4}$$

After integration, supposing that $[Cu^{II}\_1]_0 = [Cu^{II}\_2]_0 = 0$, the resulting concentration profiles for all species are (Eq. S5-S7):

$$[Cu^I] = [Cu^I]_0 e^{-k_1 t} \tag{S5}$$

$$[Cu^{II}\_1] = [Cu^I]_0 \frac{k_1}{k_2 - k_1}(e^{-k_1 t} - e^{-k_2 t}) \tag{S6}$$

$$[Cu^{II}\_2] = [Cu^I]_0(1 + \frac{k_1 e^{-k_2 t} - k_2 e^{-k_1 t}}{k_2 - k_1}) \tag{S7}$$

Knowing the values of [X](t) (where X may be $\mathbf{Cu^I}$, $\mathbf{Cu^{II}\_1}$, or $\mathbf{Cu^{II}\_2}$) from MCR, these kinetics models were employed to retrieve values of k_1 and k_2 as best fits of these curves. The resulting values were $k_1 = 0.00446$ and $k_2 = 0.00080$, obtained through a reasonably good fit ($R^2 = 0.980$). The model curves are reported in Figure S4.

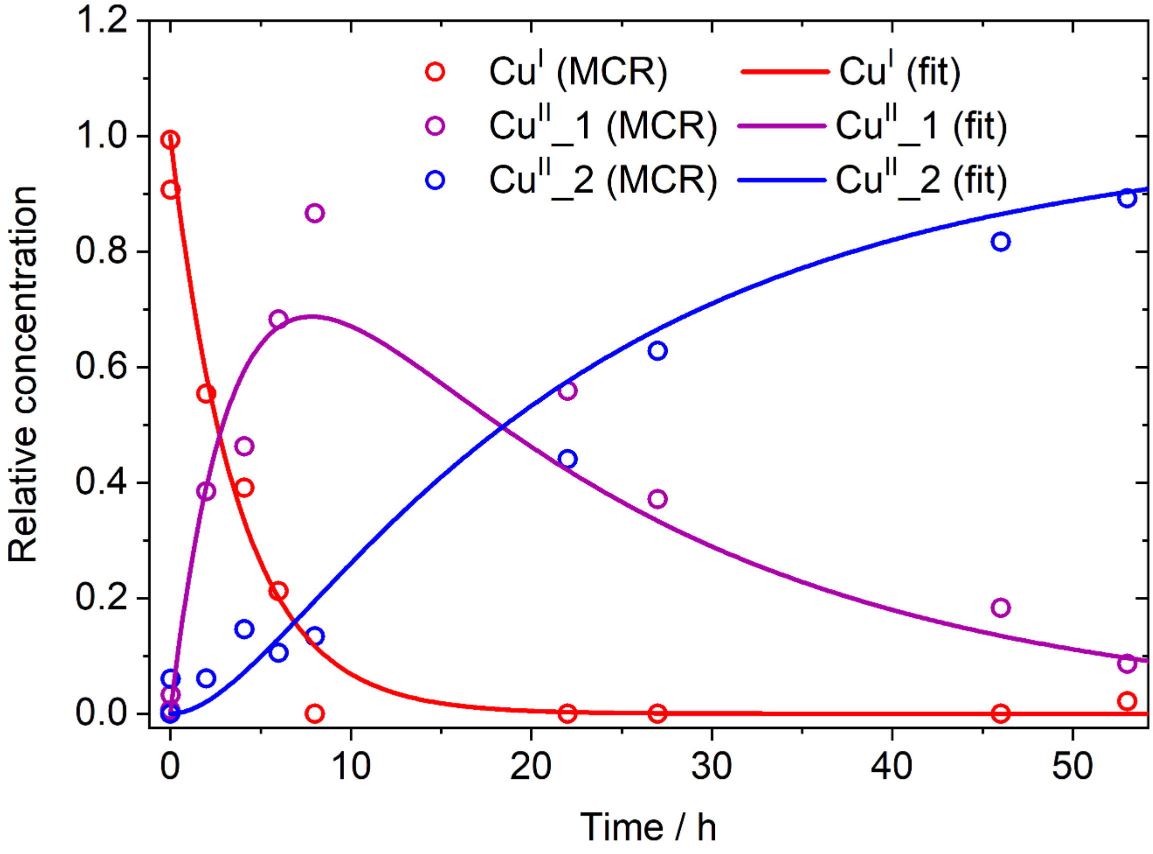

Figure S4. Best fit curves of the pure concentration profiles obtained from MCR-ALS (as reported in Figure 2c), as obtained through the procedure described in Section S4.

S5. UV-Vis spectrokinetic analysis

To retrieve more accurate information about the kinetics of these two reactions (*i.e.*, **Cu^{I}→Cu^{II}_1** and **Cu^{II}_1→Cu^{II}_2**), a spectrokinetic study was conducted exploiting the Ostwald method of flooding. Briefly, if the two known reactants have unknown reaction order, and supposing that the kinetic process is represented by a single reaction rate constant, the reaction rate can be written as in Eq. S8:

$$v(t) = k[A](t)^x[B](t)^y \quad \text{(S8)}$$

Where *v(t)* is the reaction rate for the process, k is the rate constant, *[A](t)* and *[B](t)* are the concentrations of the two reagents as a function of time, and *x* and *y* are the reaction orders for A and B, respectively. If B is present in excess in the mixture, $[B](t) \approx [B]_0$ at any given point of the reaction, and the reaction rate becomes (Eq. S9):

$$v(t) = k'[A](t)^x \quad \text{(S9)}$$

With $k' = k[B]_0^y$. By monitoring the time dependence of [A], and if the reaction is first-order in A, the equation (Eq. S10):

$$\ln[A] = -k't + \ln[A]_0 \quad \text{(S10)}$$

Eq. S3 is found to be linear and the conditional k' rate constant specific for a given $[B]_0$ can be retrieved. By varying the excess of B (*i.e.*, $[B]_0$) the reaction order y for B and the general reaction rate constant k can be calculated with a $\ln[B]_0$ vs ln(k') linear plot according Eq. S11:

$$\ln(k') = \ln(k) + yln[B]_0 \quad \text{(S11)}$$

With the slope and the exponentiation of the intercept yielding the reaction order for B and the general reaction rate constant, respectively. The two reactions were run with a tBuOOH:Cu ratio of 1, 10, 20, 60, 100 and 200 and monitored spectroscopically with UV-vis: the intensity of the

MLCT band at 21626 cm^{-1} was used to quantify the decay over time of $\mathbf{Cu^I}$ ($\mathbf{Cu^I}$→$\mathbf{Cu^{II}}$**_1**), while a two-components fit of the d-d zone of the spectrum was used to quantify the conversion $\mathbf{Cu^{II}}$**_1**→$\mathbf{Cu^{II}}$**_2**. An example of the spectral series obtained through this method is shown in Figure S5.

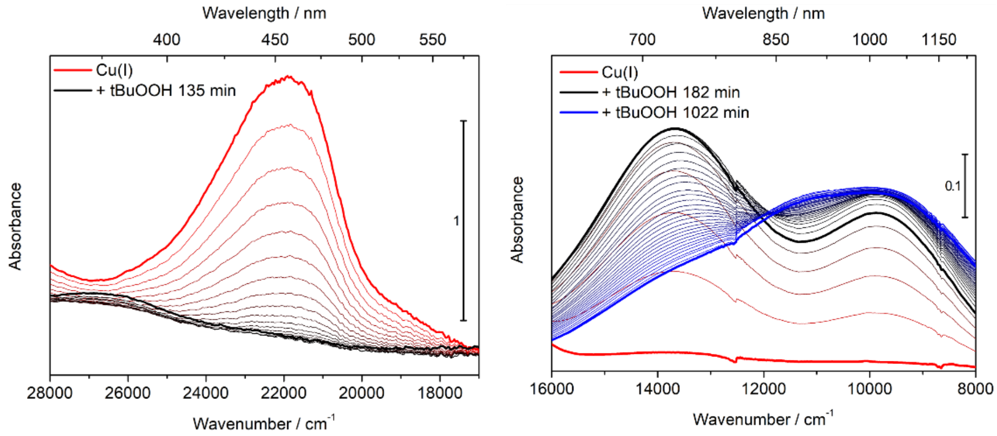

Figure S5. Example of spectrokinetic series of $\mathbf{Cu^I}$ oxidation by tBuOOH ($\mathbf{Cu^I}$:*t*BuOOH in 1:60 molar ratio)followed with in situ UV-vis spectroscopy. The starting solution of $\mathbf{Cu^I}$ was 2 mM in a 1:1 CH_3CN/CH_2Cl_2 mixture in all cases. Left panel: UV-vis spectra in the MLCT region. Right panel: UV-vis spectra in the d-d region.

While the intensity of the MLCT band at 22700 cm^{-1} could be directly used as an indicator for the concentration of $\mathbf{Cu^I}$ (and thus to monitor its conversion to $\mathbf{Cu^{II}}$**_1**), the profile of the d-d bands significantly changes between the two reactions. The spectra of $\mathbf{Cu^{II}}$**_1** and $\mathbf{Cu^{II}}$**_2** were thus fitted using two Gaussian bands per pure spectrum to obtain the parameters listed in Table S2.

Table S2. Parameters of the d-d transitions retrieved by gaussian curve fitting of spectra for the two species formed after reaction of **Cu^{I}** with tBuOOH.

Species	Band energy / cm^{-1}	Band splitting / cm^{-1}	FWHM / cm^{-1}	ε / M^{-1}cm^{-1}
**Cu^{II}_1**	13701; 9671	4030	3684; 2935	144; 76
**Cu^{II}_2**	10914; 8189	2725	3775; 2903	103; 53

These fitted spectra in the d-d region were the used to fit all intermediate states in the dataset by optimizing the weight coefficients (corresponding to relative concentrations) for each spectrum acquired during the reaction; two examples of this procedures are reported as example in Figure S6.

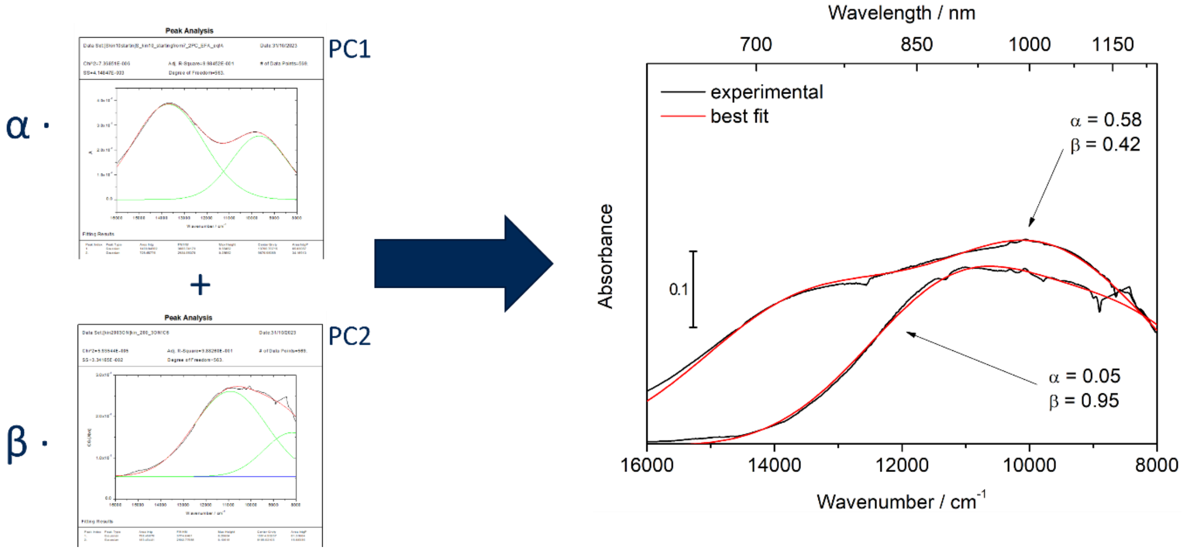

Figure S6. Example of band fitting to obtain the relative concentration of **Cu^{II}_1** and **Cu^{II}_2** for each UV-vis spectrum acquired in the spectrokinetic series of **Cu^{II}_1→Cu^{II}_2** reaction. *a* and *b* are weight coefficients (corresponding to relative concentrations) for the linear combination of

the spectra of **Cu^{II}_1** and **Cu^{II}_2**, respectively. On the right, two intermediate spectra acquired in the series are fitted according to this model, with the resulting best fit of these coefficients.

All plots of ln[A] vs t (where A = **Cu^{I}** and **Cu^{II}_1**, for reactions **Cu^{I}→Cu^{II}_1** and **Cu^{II}_1→Cu^{II}_2**, respectively) and ln$[tBuOOH]_0$ vs ln(k') displayed good linearity; data on the 1:1 proportion between complex and peroxide was not used to build this model due to the condition of $[tBuOOH](t) \approx [tBuOOH]_0$ not being valid for this composition of the solution. The kinetic parameters calculated using this model on **Cu^{I}→Cu^{II}_1** and **Cu^{II}_1→Cu^{II}_2** reactions are reported in Table S3.

Table S3. Kinetic parameters obtained for the two reactions. The reaction schemes are not in stoichiometric balance and do not necessarily include all reactants and products, but only involve the detected species in the spectrokinetic study.

Reaction	Reaction order	Rate constant
**Cu^{I}→Cu^{II}_1**	1 for **Cu^{I}**<br>0.5 for tBuOOH	$(1.4\pm0.1)\cdot10^{-3}$ $s^{-1}\cdot M^{-0.5}$
**Cu^{II}_1→Cu^{II}_2**	1 for **Cu^{II}_1**<br>0.67 for tBuOOH	$(4.0\pm0.4)\cdot10^{-4}$ $s^{-1}\cdot M^{-0.67}$

From the kinetic data obtained, both reactions appear to have a first-order dependence on the reactant containing **Cu^{I}** and **Cu^{II}_1** for **Cu^{I}→Cu^{II}_1** and **Cu^{II}_1→Cu^{II}_2** reactions, respectively, while fractional reaction orders were registered for tBuOOH in both cases, suggesting a more complex reaction mechanism involving multiple elementary steps. This agrees with i) the fact that the formation of **Cu^{II}_2** is observed even in 1:1 proportion between complex

and tBuOOH (suggesting that the complex is the limiting reagent) and ii) the absence of detectable residual organic radicals in the EPR spectra. In particular, point ii) is consistent with an overall complex:peroxide stoichiometry of 2:1 (where complex may refer to either **Cu^{I}** or **Cu^{II}_1**): supposing that homolytic cleavage of some moiety of tBuOOH is involved, the 1-electron oxidation of Cu^{I} to Cu^{II} would leave one of the free radicals in solution, possibly detectable by EPR (at least in the case of Reaction 1). Consistently with those semi-quantitatively determined by the MCR concentration profiles, the rate constants for both reactions are quite low, in a $k_1 \approx 4k_2$ proportion. The rate constant for the first reaction, involving oxidation of N-ligated Cu^{I}, is in line with similar reactions already proposed in the literature.[1] The reliability of this model, however, is based on the supposition that:

1. Reaction 1 goes to completion before reaction 2 starts (*i.e.*, [**Cu^{II}_1**]$_0$ = [**Cu^{I}**]$_0$).
2. No side reactions occur that quantitatively consume tBuOOH.
3. The concentration of tBuOOH at the start of **Cu^{II}_1→Cu^{II}_2** reaction is $[tBuOOH]_0$ - $0.5\cdot[Cu]_0$.

Although condition 1) is approximately verified, as confirmed by UV-vis spectrokinetic profiles, conditions 2) and 3) remained unchecked at this point.

S6. Spin quantification

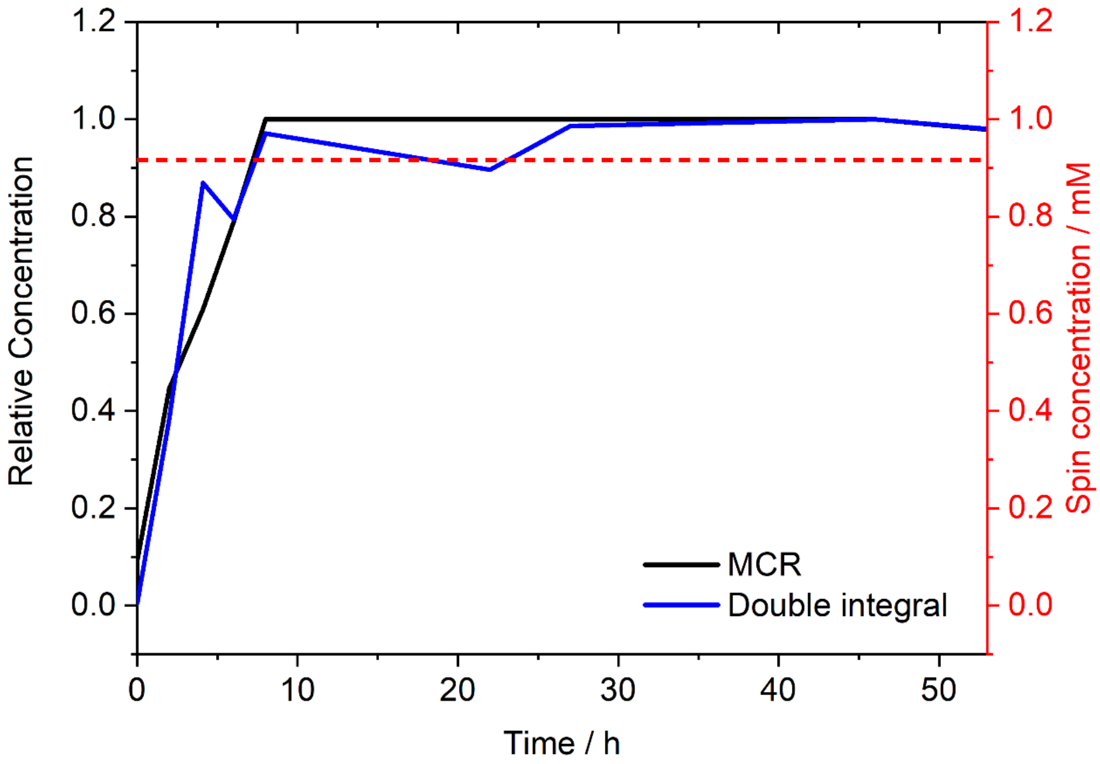

Figure S7. Comparison of concentration profiles obtained from MCR and double integral spin quantification. The relative concentration of Cu^{II} for the spin quantification procedure (left axis) was obtained by dividing the absolute amount by the total Cu concentration, while the MCR curves were obtained by summing the relative amounts of **Cu^{II}_1** and **Cu^{II}_2** in each step. The horizontal line is placed in correspondence of the total Cu concentration (right axis).

The relative amounts obtained by MCR are in line with the spin quantification curve, which is in turn consistent with the total amount of Cu within the expected experimental error (Figure S7).

The total concentration of Cu^{II} was calculated on the experimental points in which $\mathbf{Cu^{I}}$ was not present in significant amounts (*i.e.*, concentration < 1%, ca. 8 h from reaction start) by combining the double-integral of the EPR spectra, the relative proportions of the species obtained through MCR and the Aasa-Vänngård factors[2] calculated for **$\mathbf{Cu^{II}}$_1** and **$\mathbf{Cu^{II}}$_2**, according to Eq. S12:

$$C_{sa} = C_{st} \frac{J_{sa}}{J_{st}} \frac{g_{st}^{(AV)}}{g_{sa}^{(AV)}} \frac{T_{sa}}{T_{st}} \frac{MA_{st}}{MA_{st}} \sqrt{\frac{P_{st}}{P_{sa}}} \frac{f_{st}}{f_{sa}} \frac{G_{st}}{G_{sa}} \frac{SC_{st}}{SC_{sa}} \tag{S12}$$

Where C is the molar concentration, J is the double integral of the EPR signal, $g^{(VA)}$ is the Aasa-Vänngård factor for a species (a weighted average was used according to MCR concentrations), T is the temperature, MA is the modulation amplitude, P is the microwave power, f is the diameter of the EPR tube, G is the detector gain, SC is the number of scans per spectrum and the subscripts "*sa*" and "*st*" indicate sample and standard, respectively. An average deviation of 1.5% was obtained compared to the known total Cu concentration of the solution, testifying to the accuracy of this methodology to quantify Cu^{II} species in a mixture and assigning the relative concentrations.

S6. EPR fitting of $\mathbf{Cu^{II}}$_1 and $\mathbf{Cu^{II}}$_2

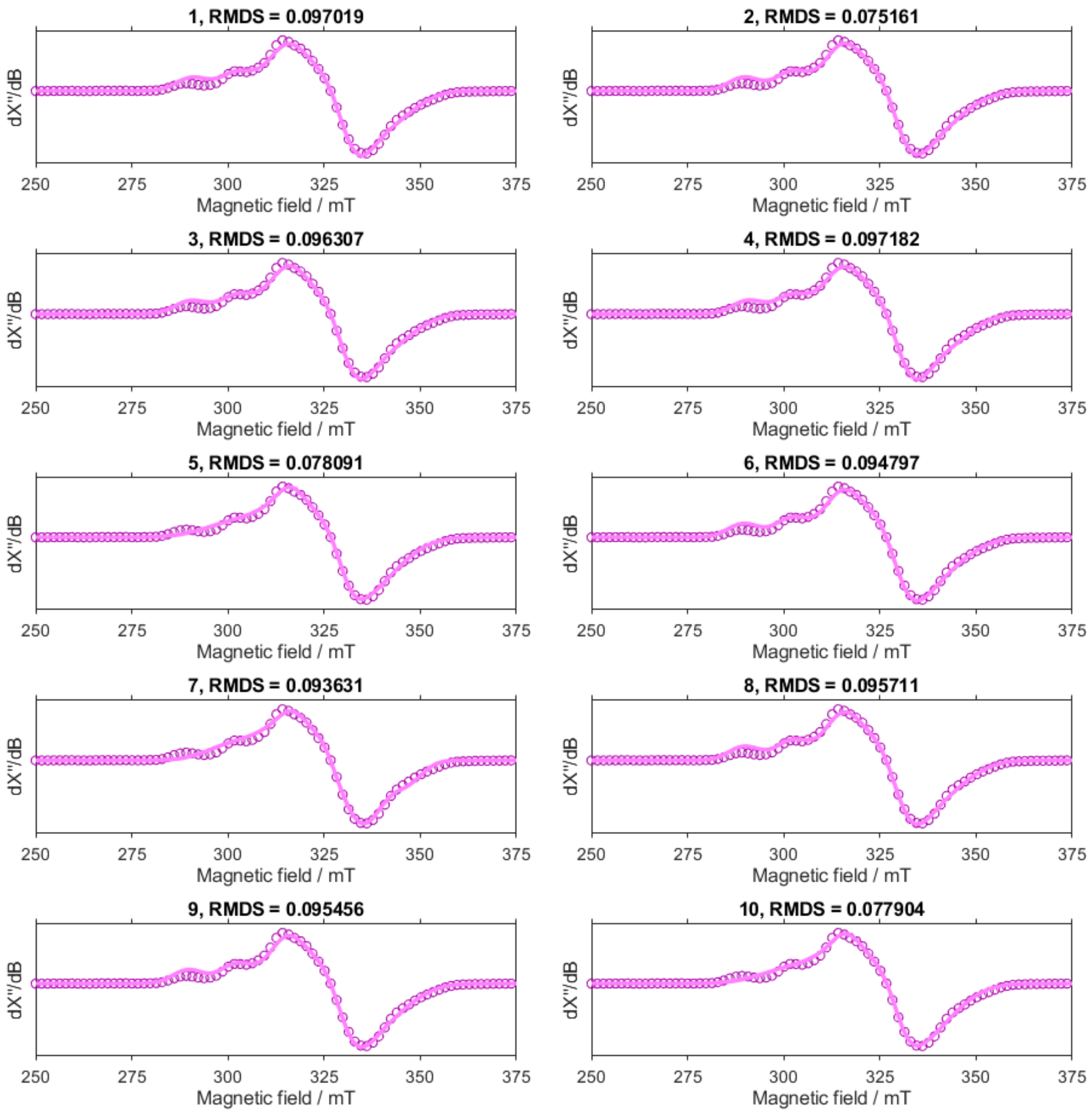

Figure S8. Experimental (empty dots) vs. fitted (full lines) EPR spectrum of **Cu^{II}_1** pure component. The fitting procedure has been repeated 10 times (AMC optimizer). In experimental spectra, only 1 point over 8 is shown for the sake of visualization. The full set of fitted parameters is provided in Table S4.

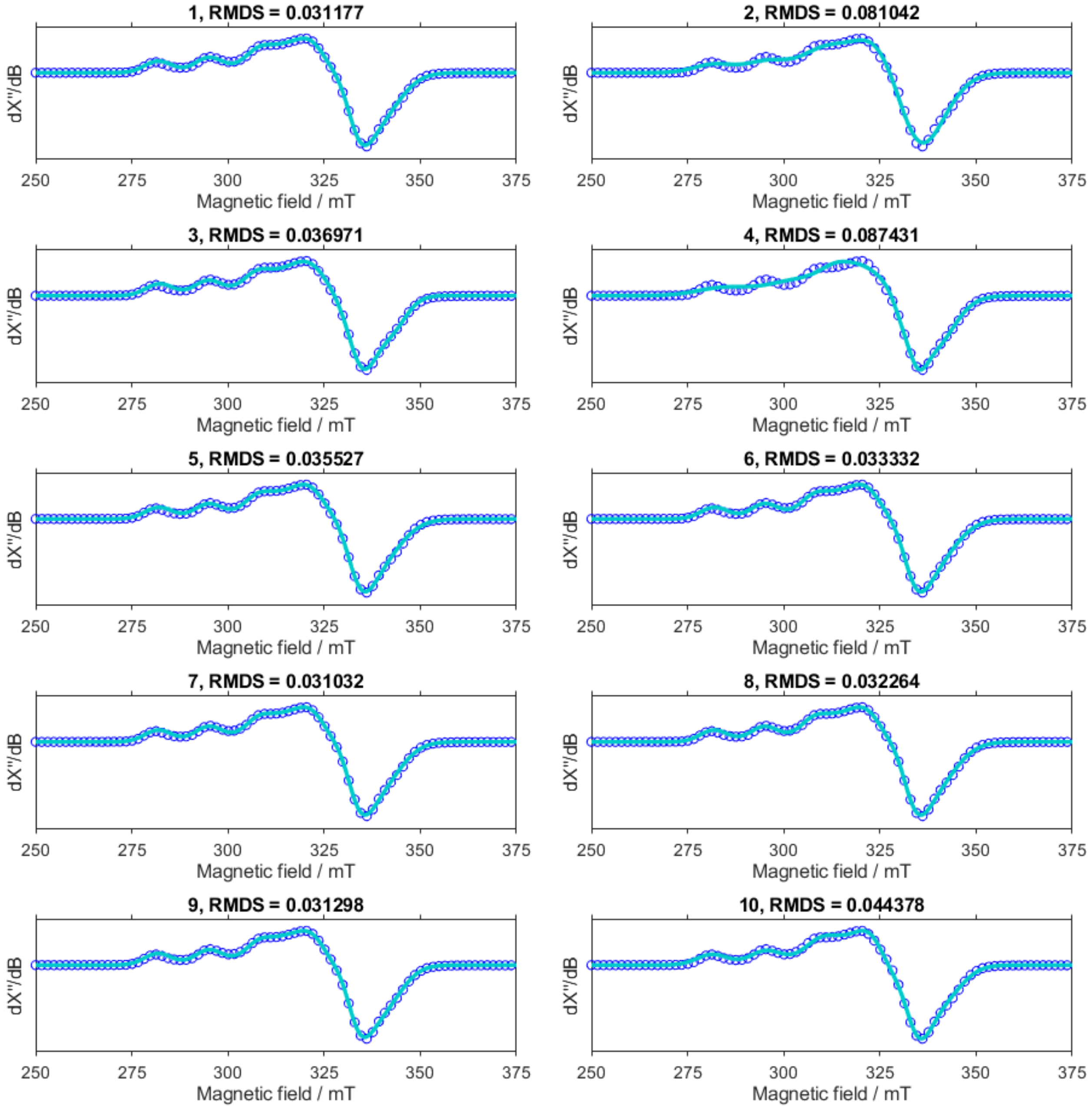

Figure S9. Experimental (empty dots) vs. fitted (full lines) EPR spectrum of **Cu^{II}_2** pure component. The fitting procedure has been repeated 10 times (AMC optimizer). In experimental spectra, only 1 point over 8 is shown for the sake of visualization. The full set of fitted parameters is provided in Table S5.

Table S4. Spin-Hamiltonian parameters obtained by 10 repeated fits (AMC optimizer) of the pure spectrum of **Cu^{II}_1** (as from MCR decomposition). Gray-backgrounded lines are outliers excluded from the calculation of averaged parameters.

#	g_x	g_y	g_z	A_x	A_y	A_z	H_x	H_y	H_z	RMSD
1	2.0514	2.1177	2.2331	192	74	359	383	287	301	0.09702
2	2.0479	2.1278	2.2305	149	2	382	460	357	292	0.07516
3	2.0502	2.1228	2.2308	178	65	371	414	295	310	0.09631
4	2.0529	2.1161	2.2340	196	74	357	374	286	300	0.09718
5	2.0449	2.1275	2.2044	111	107	295	477	197	502	0.07809
6	2.0449	2.1305	2.2308	128	1	385	491	352	292	0.09480
7	2.0665	2.0955	2.2098	252	45	268	272	211	432	0.09363
8	2.0481	2.1271	2.2301	157	10	383	457	352	292	0.09571
9	2.0472	2.1281	2.2304	148	11	383	462	351	289	0.09546
10	2.0451	2.1274	2.2043	108	107	296	483	199	501	0.07790

Table S5. Spin-Hamiltonian parameters obtained by 10 repeated fits (AMC optimizer) of the pure spectrum of **Cu^{II}_2** (as from MCR decomposition). Gray-backgrounded lines are outliers excluded from the calculation of averaged parameters.

#	g_x	g_y	g_z	A_x	A_y	A_z	H_x	H_y	H_z	RMSD
1	2.0500	2.1208	2.2827	96	135	412	382	218	264	0.03118
2	2.0900	2.0917	2.2748	198	3	433	422	292	387	0.08104
3	2.0826	2.0866	2.2858	234	1	398	273	205	263	0.03697
4	2.0728	2.1479	2.2235	41	108	527	135	253	505	0.08743
5	2.0484	2.1209	2.2837	23	143	404	456	220	265	0.03553
6	2.0496	2.1203	2.2829	73	139	408	422	220	264	0.03333
7	2.0499	2.1209	2.2828	96	135	412	378	218	263	0.03103
8	2.0497	2.1207	2.2832	85	137	410	404	219	263	0.03226
9	2.0503	2.1215	2.2826	110	133	414	337	214	263	0.03130
10	2.0791	2.0989	2.2770	217	51	438	345	192	298	0.04438

S7. DFT structures

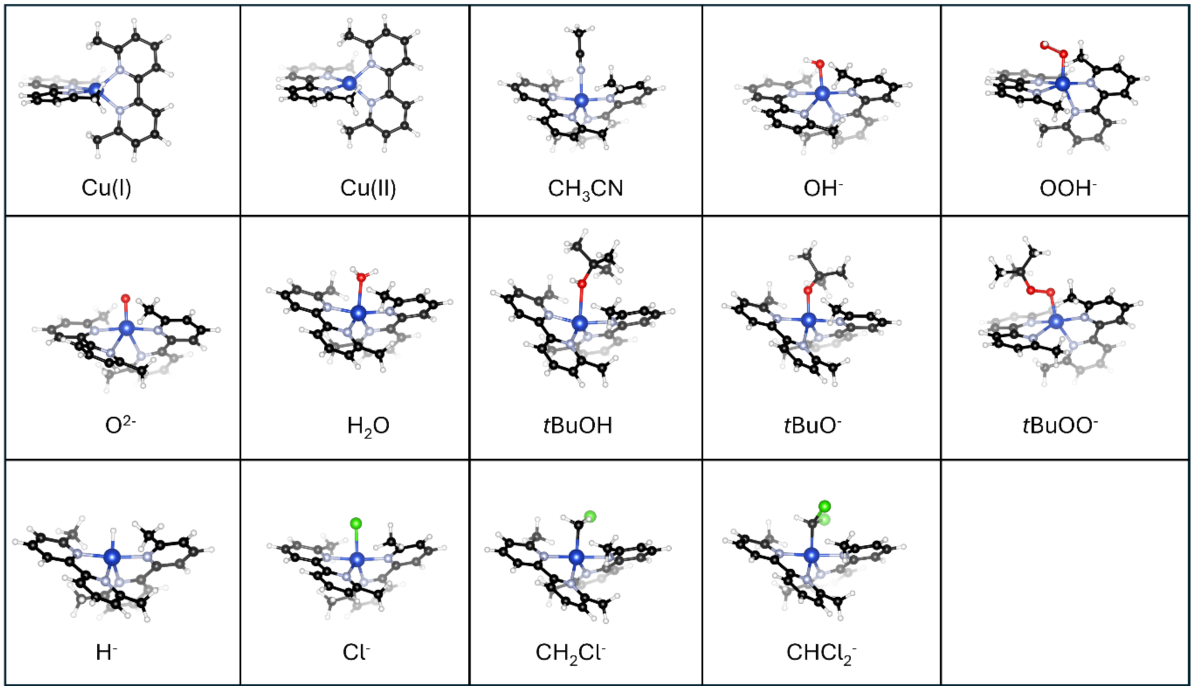

Figure S10. Structural models used for the calculation of spectroscopic properties; geometries in .xyz format are reported in Appendix II. Color code: H in white; C in black; N in light blue; O in red; Cl in green; Cu in blue.

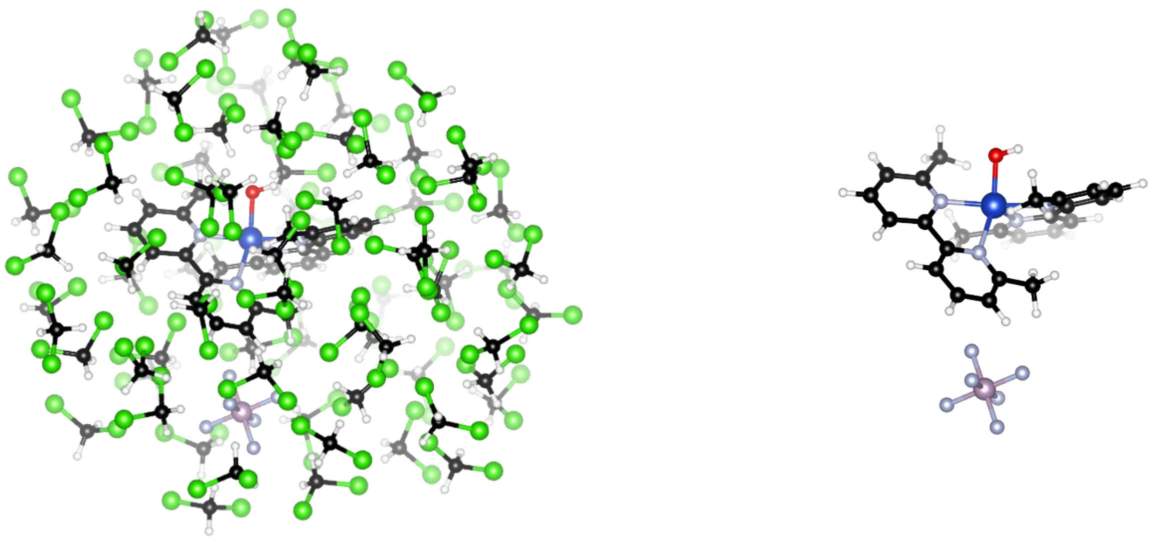

Figure S11. Example structural model for the ONIOM optimized structure of [Cu^{II}(6,6'-dimethyl-2,2'-bipyridyl)$_2$(OH)]PF_6 in its solvation shell (left) and with PF_6^- anion only (right). Color code: H in white; C in black; N in light blue; O in red; Cl in green; Cu in blue.

Table S6. Spin-Hamiltonian parameters obtained from DFT calculations for [Cu^{II}(6,6'-dimethyl-2,2'-bipyridyl)$_2$(OH)]$^+$, [Cu^{II}(6,6'-dimethyl-2,2'-bipyridyl)$_2$(OH)]PF_6 and [Cu^{II}(6,6'-dimethyl-2,2'-bipyridyl)$_2$(OH)]PF_6 + 70 CH_2Cl_2 molecules.

Model	g_x	g_y	g_z	A_x	A_y	A_z
[Cu^{II}(6,6'-dimethyl-2,2'-bipyridyl)$_2$(OH)]$^+$	2.0594	2.0855	2.2603	50	63	569
[Cu^{II}(6,6'-dimethyl-2,2'-bipyridyl)$_2$(OH)]PF_6	2.0585	2.0861	2.2541	1	70	553
[Cu^{II}(6,6'-dimethyl-2,2'-bipyridyl)$_2$(OH)]PF_6+70CH_2Cl_2	2.0647	2.0844	2.2666	18	62	554

S8. Extended experimental and computational methods

Compounds synthesis

Synthesis of [Cu^{I}(6,6'-dimethyl-2,2'-bipyridyl)$_2$]PF_6,[3] [Cu^{II}(6,6'-dimethyl-2,2'-bipyridyl)$_2$(H_2O)]$(OTf)_2$ (OTf = triflate)[4] and [Cu^{II}(6,6'-dimethyl-2,2'-bipyridyl)$_2$]$(ClO_4)_2$[5] was performed according to literature procedures. Briefly, [Cu^{I}(6,6'-dimethyl-2,2'-bipyridyl)$_2$]PF_6 was prepared by dissolution of [$Cu^{I}(CH_3CN)_4$]PF_6 and the ligand in ca. 1:2 proportion in CH_2Cl_2; stirring of the solution for 3 h at room temperature in inert atmosphere led to quantitative yields of the complex after filtration. [Cu^{II}(6,6'-dimethyl-2,2'-bipyridyl)$_2$(H_2O)]$(OTf)_2$ was prepared by mixing a solution of $Cu^{II}(OTf)_2$ in CH_2Cl_2 with a solution of 6,6'-dimethyl-2,2'-bipyridine in CH_2Cl_2 (1:2.2 molar ratio) in a reaction flask. The liquid immediately changed from a blue solution to a green suspension, and after 1 h of stirring at room temperature in air the precipitate was filtrated, washed twice with diethyl ether and recovered as a light green powder in quantitative yield. The use of a chloride salt ($Cu^{II}Cl_2$) as a metal source was avoided due to the possibility of the Cl^- anion to enter the first coordination sphere of the metal.[6] [Cu^{II}(6,6'-dimethyl-2,2'-bipyridyl)$_2$]$(ClO_4)_2$ was prepared by adding 6,6'-dimethyl-2,2'-bipyridine in an aqueous solution of $Cu^{II}(ClO_4)_2$ hexahydrate (1:2 molar ratio) and agitating vigorously for 1 h. An excess of $KClO_4$ was added as a solid and the resulting precipitate was filtered and washed with cold water. The resulting red/brown powder was dried at 50 °C *in vacuo* for 1 h and stored in a Schlenk flask to avoid contact with air moisture. Due to the increased polarity of this compound compared to its Cu^{I} analogue, its solubility in CH_2Cl_2 is very poor; CH_3NO_2 was thus employed as a polar, non-coordinating solvent to dissolve it, obtaining a pale red solution. By contrast, dissolution of [Cu^{II}(6,6'-dimethyl-2,2'-

bipyridyl)$_2$](ClO$_4$)$_2$ in anhydrous CH_3CN results in an immediate change in color (from red/brown to pale yellow/green), ascribed to the formation of [Cu^{II}(dmbp)$_2$(CH_3CN)](ClO$_4$)$_2$.

EPR spectroscopy

Saturation effects during the collection of EPR spectra were excluded by checking linearity between double integral of the spectrum and square root of power in a power series of a 1:1 CH_3CN/CH_2Cl_2 1 mM solution of [Cu^{II}(6,6'-dimethyl-2,2'-bipyridyl)$_2$(CH_3CN)](ClO$_4$)$_2$ in the range of 0.05-0.47 mW; the power used in all experiments was set to 0.19 mW. The power saturation curve is reported in Figure S12.

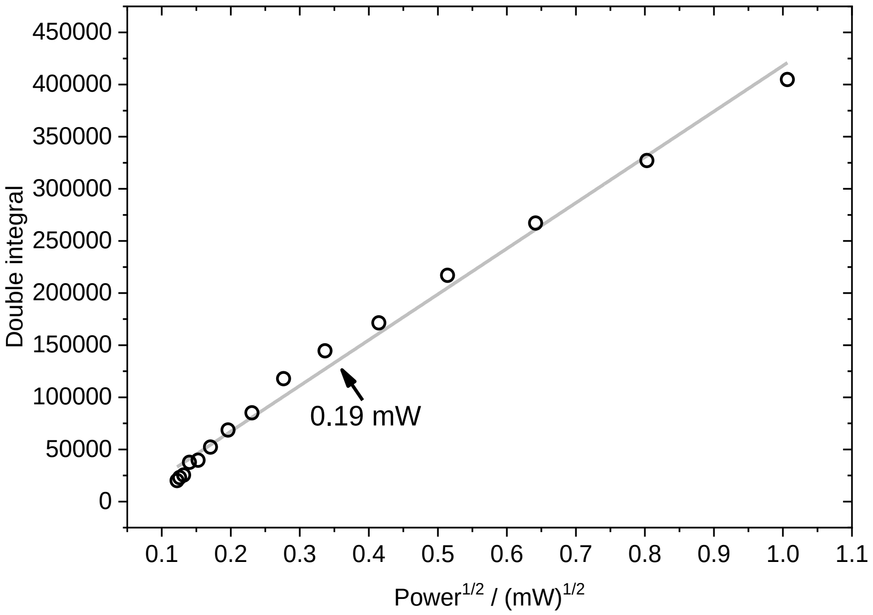

Figure S12. Double integral of the EPR signal of a 1 mM solution of Cu^{II}(6,6'-dimethyl-2,2'-bipyridyl)$_2$(CH_3CN)](ClO$_4$)$_2$ as a function of the square root of the radiation power. The point marked with the arrow corresponds to the conditions used for the spectra collected on the oxidation series (power = 0.19 mW). The grey line represents a linear fit of the data points.

DFT calculations

Density functional theory (DFT) calculations were performed with the ORCA 5.0.3 code.[7–9] All structures were optimized using the hybrid B3LYP functional[10,11] and the def2-TZVP basis set developed by Ahlrichs and coworkers.[12] The def2/J basis sets[13] along with chain of spheres approximation (RIJCOSX) were used for the RI (resolution of identity) approximation to the Coulomb integrals.[14] Dispersive forces were included in the calculation through the Grimme D3 empirical scheme with Becke–Johnson damping.[15] The effect of solvation was implicitly accounted for via the polarizable conductor calculation model (CPCM) method.[16,17] PF_6^- as a counterion was not included in the calculations, since both DFT and multi-level optimizations that included it (including explicit solvent molecules in the latter case) resulted in equivalent spectral features to the implicitly-solvated complex cation (see Table S6). Initial models were geometrically optimized, and vibrational frequencies were computed; the absence of imaginary frequencies confirmed that minimum structures were obtained. Multi-level geometry optimizations were performed according to the following scheme. A solvating shell of 70 CH_2Cl_2 molecules was generated around the DFT-optimized structure of the complex (which was kept fixed) using the QCG cluster growth algorithm[18] provided in the CREST software.[19] The resulting metadynamics-generated ensemble of solvated structures,[20] generated at the GFN-FF level,[21] was energy-screened; the two most stable structures were found to account for >95% of a Boltzmann population. The minimum energy conformer was further reoptimized using a combined DFT/GFN2-xTB[22] ONIOM scheme as implemented in the ORCA software with standard xTB settings (for the solvent molecules) and identical DFT settings as the one employed for classical optimization procedures described above (apart for implicit solvation, which was

excluded). EPR parameters were calculated on the resulting optimized structures as for the regular DFT-obtained structures (Table S6).

REFERENCES

(1) Elwell, C. E.; Gagnon, N. L.; Neisen, B. D.; Dhar, D.; Spaeth, A. D.; Yee, G. M.; Tolman, W. B. Copper-Oxygen Complexes Revisited: Structures, Spectroscopy, and Reactivity. *Chem. Rev.* **2017**, *117* (3), 2059–2107. https://doi.org/10.1021/ACS.CHEMREV.6B00636/.

(2) Aasa, R.; Vänngård, tore. EPR Signal Intensity and Powder Shapes: A Reexamination. *J. Magn. Reson.* **1975**, *19* (3), 308–315. https://doi.org/10.1016/0022-2364(75)90045-1.

(3) Fresta, E.; Volpi, G.; Milanesio, M.; Garino, C.; Barolo, C.; Costa, R. D. Novel Ligand and Device Designs for Stable Light-Emitting Electrochemical Cells Based on Heteroleptic Copper(I) Complexes. *Inorg. Chem.* **2018**, *57* (16), 10469–10479. https://doi.org/10.1021/ACS.INORGCHEM.8B01914.

(4) Hall, J. R.; Litzow, M. R.; Plowman, R. A. Coordination Compounds of Substituted 1,10-Phenanthrolines and Related Dipyridyls. VI. Complexes of Copper(II) and 4,6,4’,6’-Tetramethyl-2,2’-Bipyridine. *Aust. J. Chem.* **1965**, *18* (9), 1331–1338. https://doi.org/10.1071/CH9651331.

(5) Munakata, M.; Kitagawa, S.; Asahara, A.; Masuda, H. Crystal Structure of Bis(2,2′-Bipyridine)Copper(I) Perchlorate. *https://doi.org/10.1246/bcsj.60.1927* **2006**, *60* (5), 1927–1929. https://doi.org/10.1246/BCSJ.60.1927.

(6) Giordano, M.; Volpi, G.; Bonomo, M.; Mariani, P.; Garino, C.; Viscardi, G. Methoxy-Substituted Copper Complexes as Possible Redox Mediators in Dye-Sensitized Solar Cells. *New J. Chem.* **2021**, *45* (34), 15303–15311. https://doi.org/10.1039/D1NJ02577E.

(7) Neese, F.; Wiley, J. The ORCA Program System. *Wiley Interdiscip. Rev. Comput. Mol. Sci.* **2012**, *2* (1), 73–78. https://doi.org/10.1002/WCMS.81.

(8) Neese, F.; Wennmohs, F.; Becker, U.; Riplinger, C. The ORCA Quantum Chemistry Program Package. *J. Chem. Phys.* **2020**, *152* (22), 224108. https://doi.org/10.1063/5.0004608/1061982.

(9) Neese, F. Software Update: The ORCA Program System—Version 5.0. *Wiley Interdiscip. Rev. Comput. Mol. Sci.* **2022**, *12* (5), e1606. https://doi.org/10.1002/WCMS.1606.

(10) Becke, A. D. A New Mixing of Hartree–Fock and Local Density- functional Theories. *J. Chem. Phys.* **1993**, *98* (2), 1372–1377. https://doi.org/10.1063/1.464304.

(11) Lee, C.; Yang, W.; Parr, R. G. Development of the Colle-Salvetti Correlation-Energy Formula into a Functional of the Electron Density. *Phys. Rev. B* **1988**, *37* (2), 785. https://doi.org/10.1103/PhysRevB.37.785.

(12) Weigend, F.; Ahlrichs, R. Balanced Basis Sets of Split Valence, Triple Zeta Valence and Quadruple Zeta Valence Quality for H to Rn: Design and Assessment of Accuracy. *Phys. Chem. Chem. Phys.* **2005**, *7* (18), 3297–3305. https://doi.org/10.1039/B508541A.

(13) Weigend, F. Accurate Coulomb-Fitting Basis Sets for H to Rn. *Phys. Chem. Chem. Phys.* **2006**, *8* (9), 1057–1065. https://doi.org/10.1039/B515623H.

(14) Helmich-Paris, B.; de Souza, B.; Neese, F.; Izsák, R. An Improved Chain of Spheres for Exchange Algorithm. *J. Chem. Phys.* **2021**, *155* (10). https://doi.org/10.1063/5.0058766.

(15) Grimme, S.; Ehrlich, S.; Goerigk, L. Effect of the Damping Function in Dispersion

Corrected Density Functional Theory. *J. Comput. Chem.* **2011**, *32* (7), 1456–1465. https://doi.org/10.1002/JCC.21759.

(16) Barone, V.; Cossi, M. Quantum Calculation of Molecular Energies and Energy Gradients in Solution by a Conductor Solvent Model. *J. Phys. Chem. A* **1998**, *102* (11), 1995–2001. https://doi.org/10.1021/JP9716997/ASSET/IMAGES/LARGE/JP9716997F00004.JPEG.

(17) Cossi, M.; Rega, N.; Scalmani, G.; Barone, V. Energies, Structures, and Electronic Properties of Molecules in Solution with the C-PCM Solvation Model. *J. Comput. Chem.* **2003**, *24* (6), 669–681. https://doi.org/10.1002/JCC.10189.

(18) Spicher, S.; Plett, C.; Pracht, P.; Hansen, A.; Grimme, S. Automated Molecular Cluster Growing for Explicit Solvation by Efficient Force Field and Tight Binding Methods. *J. Chem. Theory Comput.* **2022**, *18*, 3189. https://doi.org/10.1021/ACS.JCTC.2C00239.

(19) Pracht, P.; Bohle, F.; Grimme, S. Automated Exploration of the Low-Energy Chemical Space with Fast Quantum Chemical Methods. *Phys. Chem. Chem. Phys.* **2020**, *22* (14), 7169–7192. https://doi.org/10.1039/C9CP06869D.

(20) Grimme, S. Exploration of Chemical Compound, Conformer, and Reaction Space with Meta-Dynamics Simulations Based on Tight-Binding Quantum Chemical Calculations. *J. Chem. Theory Comput.* **2019**, *15* (5), 2847–2862. https://doi.org/10.1021/ACS.JCTC.9B00143.

(21) Spicher, S.; Grimme, S. Robust Atomistic Modeling of Materials, Organometallic, and Biochemical Systems. *Angew. Chemie Int. Ed.* **2020**, *59* (36), 15665–15673. https://doi.org/10.1002/ANIE.202004239.

(22) Bannwarth, C.; Ehlert, S.; Grimme, S. GFN2-XTB - An Accurate and Broadly Parametrized Self-Consistent Tight-Binding Quantum Chemical Method with Multipole

Electrostatics and Density-Dependent Dispersion Contributions. *J. Chem. Theory Comput.* **2019**, *15* (3), 1652–1671. https://doi.org/10.1021/ACS.JCTC.8B01176.

Appendix I: AMC Matlab® script

The following Matlab® script allows for AMC fitting of a single-component EPR spectrum, considering 9 free parameters (g_x, g_y, g_z, A_x, A_y, A_z, $HStrain_x$, $HStrain_y$, $HStrain_z$). Experimental data must be supplied as a *N*x*1* column vector of intensities (where *N* is the number of datapoints). The script requires the Easyspin (tested with version 6.0.4) executables are found in the Matlab® path. For parallel usage, the Matlab® Parallel Toolbox must be installed as well.

```
%% fitting control parameters
tolfun=1e-5;      %convergence criterion for RMSD
n=300;      %number of MC trials per cycle
ad=0.02;          %range adaption coefficients (scales RMSD)

%%parameter ranges for initial guess
gxmin=2.0;
gxmax=2.3;
gymin=2.0;
gymax=2.3;
gzmin=2.0;
gzmax=2.3;

Axmin=0;
Axmax=500;
Aymin=0;
Aymax=500;
Azmin=0;
Azmax=500;

Hxmin=1;
Hxmax=500;
Hymin=1;
```

```
Hymax=500;
Hzmin=1;
Hzmax=500;

datanorm=norm(data);

%% initial guess
tic
gxguess=gxmin+rand*(gxmax-gxmin);
gyguess=gymin+rand*(gymax-gymin);
if gxguess > gyguess
    gzguess=gxguess+rand*(gzmax-gxguess);
else
    gzguess=gyguess+rand*(gzmax-gyguess);
end

Axguess=Axmin+rand*(Axmax-Axmin);
Ayguess=Aymin+rand*(Aymax-Aymin);
if Axguess > Ayguess
    Azguess=Axguess+rand*(Azmax-Axguess);
else
    Azguess=Ayguess+rand*(Azmax-Ayguess);
end

Hxguess=Hxmin+rand*(Hxmax-Hxmin);
Hyguess=Hymin+rand*(Hymax-Hymin);
Hzguess=Hzmin+rand*(Hzmax-Hzmin);

mi=1;

Exp = struct();
Exp.mwFreq = 9.638051;
Exp.Range=[250 375];
Exp.nPoints = 640;
Exp.Temperature = 30;
Exp.Harmonic = 1;
SysCu = struct();
SysCu.Nucs = 'Cu';
```

```
SysCu.g = [gxguess gyguess gzguess];
SysCu.A = [Axguess Ayguess Azguess];
SysCu.HStrain = [Hxguess Hyguess Hzguess];
sim=pepper(SysCu,Exp);
output=[SysCu.g SysCu.A SysCu.HStrain rms(sim*datanorm/norm(sim)-data')];
rmsdnew=output(mi,10);
rmsd=rmsdnew+1;
i=0;
term=0;
term_var=0;

%% fitting routine
while rmsd > tolfun
    drmsd=abs(rmsd-rmsdnew);
    if drmsd < tolfun/1000
        term_var=term_var+1;
        if term_var==3
            disp(['RMSD variation less than ' num2str(tolfun) ' for 3
consecutive cycles, interrupting'])
            break
        end
    else
        term_var=0;
    end
    if rmsdnew < rmsd
        rmsd=rmsdnew;
        term=0;
        disp(['RMSD at cycle ',num2str(i),': ',num2str(rmsd)])
        gxmin=output(mi,1)-rmsd*ad;
        gxmax=output(mi,1)+rmsd*ad;
        gymin=output(mi,2)-rmsd*ad;
        gymax=output(mi,2)+rmsd*ad;
        gzmin=output(mi,3)-rmsd*ad;
        gzmax=output(mi,3)+rmsd*ad;


        Axmin=output(mi,4)-rmsd*1000*ad;
        if Axmin < 0
```

```
        Axmin=0;
    end
    Axmax=output(mi,4)+rmsd*1000*ad;
    Aymin=output(mi,5)-rmsd*1000*ad;
    if Aymin < 0
        Axmin=0;
    end
    Aymax=output(mi,5)+rmsd*1000*ad;
    Azmin=output(mi,6)-rmsd*1000*ad;
    if Azmin < 0
        Axmin=0;
    end
    Azmax=output(mi,6)+rmsd*1000*ad;

    Hxmin=output(mi,7)-rmsd*1000*ad;
    if Hxmin < 0
        Hxmin=10;
    end
    Hxmax=output(mi,7)+rmsd*1000*ad;
    Hymin=output(mi,8)-rmsd*1000*ad;
    if Hymin < 0
        Hymin=10;
    end
    Hymax=output(mi,8)+rmsd*1000*ad;
    Hzmin=output(mi,9)-rmsd*1000*ad;
    if Hzmin < 0
        Hzmin=10;
    end
    Hzmax=output(mi,9)+rmsd*1000*ad;
else
    disp(['RMSD not improving at cycle ',num2str(i)])
    term=term+1;
end
if term==10
    disp('RMSD not improving for 10 consecutive cycles, interrupting')
    break
end
```

```
    output=[];


    parfor j=1:round(n/rmsd)    %change "parfor" to "for" for a serial execution
        Exp = struct();
        Exp.mwFreq = 9.638051;
        Exp.Range=[250 375];
        Exp.nPoints = 640;
        Exp.Temperature = 30;
        Exp.Harmonic = 1;
        SysCu = struct();
        SysCu.Nucs = 'Cu'
        SysCu.g = [gxmin+rand*(gxmax-gxmin) gymin+rand*(gymax-gymin) gzmin+rand*(gzmax-gzmin)];
        SysCu.A = [Axmin+rand*(Axmax-Axmin) Aymin+rand*(Aymax-Aymin) Azmin+rand*(Azmax-Azmin)];
        SysCu.HStrain = [Hxmin+rand*(Hxmax-Hxmin) Hymin+rand*(Hymax-Hymin) Hzmin+rand*(Hzmax-Hzmin)];
        sim=pepper(SysCu,Exp);
        output=[output;[SysCu.g SysCu.A SysCu.HStrain rms(sim*datanorm/norm(sim)-data')]];
    end
    [rmsdnew,mi]=min(output(:,10));
    i=i+1;

end


%% retrieve optimized parameters

[rmsd,mi]=min(output(:,10));
[~,I]=sort(output(mi,1:3));
output(mi,:)=[output(mi,I(1)) output(mi,I(2)) output(mi,I(3)) output(mi,I(1)+3) output(mi,I(2)+3) output(mi,I(3)+3) output(mi,I(1)+6) output(mi,I(2)+6) output(mi,I(3)+6) output(mi,10)];
Exp = struct();
Exp.mwFreq = 9.638051;
```

```
Exp.Range=[250 375];
Exp.nPoints = 640;
Exp.Temperature = 30;
Exp.Harmonic = 1;
SysCu = struct();
SysCu.Nucs = 'Cu';
SysCu.g = [output(mi,1) output(mi,2) output(mi,3)];
SysCu.A = [output(mi,4) output(mi,5) output(mi,6)];
SysCu.HStrain = [output(mi,7) output(mi,8) output(mi,9)];

B=linspace(250,375,640)';
sim=pepper(SysCu,Exp);
plot(B,data,'o',B,sim*datanorm/norm(sim))
toc
```

Appendix II: .xyz optimized DFT geometries

CuI

53
CuI

H	-4.90873110956837	-0.89434706459854	0.51594603556152
C	-2.59206657721708	-2.72706626774958	2.15007906448105
H	-2.48508594669355	-3.56902457381919	2.82030487405357
C	-3.84336877318494	-2.31033222462326	1.72095062574668
H	-4.73604955339092	-2.82694021217739	2.04898246218416
C	-3.94195572429415	-1.22470293763404	0.86424791040959
C	-2.77562163635650	-0.58288354067481	0.45797112474652
N	-1.56622645230077	1.00100142415925	-0.87718680017404
C	-1.45932849370452	2.04916453099311	-1.70675630518355
C	-0.07856524371217	2.45422690991879	-2.11863390518925
H	-0.10738565016816	3.23564021716556	-2.87619210195418
H	0.47851078856117	2.82295373539077	-1.25430535274129
H	0.46740085296083	1.59312042009465	-2.50857156082754
N	-1.56626592656306	-1.00088107941081	0.87715209218210
C	-1.45941335545550	-2.04904227536291	1.70672914353037
C	-0.07866341188144	-2.45420259795436	2.11855663873276
H	0.46737363728331	-1.59313715359541	2.50848570625746
H	0.47835927707640	-2.82295729377622	1.25420597261449
H	-0.10751150411288	-3.23562136976566	2.87610811749848
C	-2.59194865943372	2.72727984137496	-2.15005088863196
H	-2.48493169983200	3.56923829703671	-2.82027071624158
C	-3.84326478646911	2.31063406424849	-1.72087801388551
H	-4.73591972087599	2.82731355798392	-2.04886748885757
C	-3.94189877566492	1.22500147914010	-0.86418471644498
C	-2.77559681884761	0.58309118266566	-0.45795985169441
H	-4.90868503327934	0.89471467657268	-0.51584734886434
H	4.90873937721533	0.89434231230029	0.51588779820637
C	2.59210236010127	2.72706905183844	2.15005187449039
H	2.48513292788490	3.56902871514026	2.82027766052163
C	3.84339774426797	2.31033278467760	1.72090537506416
H	4.73608398628058	2.82694094019529	2.04892202857782
C	3.94197020549867	1.22470059008884	0.86420462312087
C	2.77562915060894	0.58288120765934	0.45794790088973
N	1.56621360871922	-1.00098837840466	-0.87720880231975
C	1.45930176418874	-2.04915459180758	-1.70677269342752
C	0.07853333280250	-2.45419697045440	-2.11865343345577
H	0.10734388930228	-3.23560441720873	-2.87621807538879
H	-0.47854637091701	-2.82292498699270	-1.25432765506887

H	-0.46742276522987	-1.59308054426850	-2.50858267446201
N	1.56627997506500	1.00088182843295	0.87714489158689
C	1.45944155299015	2.04904509649378	1.70672118948406
C	0.07869841791250	2.45420584802151	2.11857095025706
H	-0.46733078664844	1.59314124558981	2.50851291984715
H	-0.47834014215473	2.82295636541235	1.25422868868032
H	0.10755888857402	3.23562746288232	2.87611904102457
C	2.59191247212975	-2.72729256172728	-2.15005689972210
H	2.48488431465464	-3.56925326027212	-2.82027204541101
C	3.84323398692064	-2.31066490282466	-1.72088201980422
H	4.73588152393882	-2.82736211400431	-2.04886368088438
C	3.94188258777768	-1.22502840140918	-0.86419520078815
C	2.77558945496482	-0.58309618762318	-0.45797951550021
H	4.90867266517779	-0.89475566450576	-0.51585484075805
Cu	0.00000015559879	0.00000916536776	-0.00005533176871

<u>CuII</u>

53
CuII

H	-4.84386857650970	-0.79418256487657	0.67214261530484
C	-2.49993936049618	-2.80308717822650	2.03986482676443
H	-2.38081147131027	-3.65859475985305	2.68968864083449
C	-3.75609702509616	-2.32066304586086	1.71647464836579
H	-4.64386784947507	-2.80177658517406	2.10511266979100
C	-3.87171608941897	-1.19936186517566	0.90658856844396
C	-2.71658314433563	-0.60696143240635	0.42034087071817
N	-1.50014672356963	1.11375315479920	-0.71693664266227
C	-1.36667768671854	2.17294299042886	-1.52926336619233
C	0.00975255400574	2.64618347318553	-1.87437806174948
H	-0.02543996034229	3.29235908115722	-2.74913184221221
H	0.44356651792344	3.21881474690493	-1.05258120040294
H	0.67134400256722	1.80478745009991	-2.08131653311681
N	-1.50024300699238	-1.11379206964031	0.71693987661120
C	-1.36688524480227	-2.17286717908124	1.52944846757190
C	0.00948860881982	-2.64602285097643	1.87491949372313
H	0.67101778876649	-1.80458223198323	2.08186716375625
H	0.44348807621773	-3.21877100682898	1.05331201068828
H	-0.02587014151916	-3.29206026930057	2.74976852222196
C	-2.49966141845642	2.80326630455115	-2.03971130228390
H	-2.38044426929055	3.65888028510579	-2.68937853094770
C	-3.75586523931748	2.32082858441116	-1.71651660371117
H	-4.64358512932836	2.80203564662227	-2.10515526519210
C	-3.87159713931183	1.19945319831690	-0.90674552721199
C	-2.71652676080034	0.60695306183553	-0.42047224521180

H	-4.84378616226990	0.79431109192906	-0.67238739279153
H	4.84384724655661	0.79401914975320	0.67227908575928
C	2.49986560602133	2.80310010929522	2.03966503066049
H	2.38071125993137	3.65871472912107	2.68934305356863
C	3.75603518706129	2.32059142376275	1.71644148644125
H	4.64379076234863	2.80174183797516	2.10506889910150
C	3.87168605776345	1.19921667855195	0.90665774857955
C	2.71657115226967	0.60678726824240	0.42040309621982
N	1.50016153164081	-1.11388973025865	-0.71697995976620
C	1.36672735529548	-2.17296219135574	-1.52947932805997
C	-0.00967907648258	-2.64604575421802	-1.87491695918941
H	0.02562357140297	-3.29207593553073	-2.74977371432089
H	-0.44368230321383	-3.21878310064389	-1.05330384651622
H	-0.67117340863145	-1.80457106780456	-2.08183679444167
N	1.50022730309022	1.11365878299669	0.71689298956147
C	1.36683552254105	2.17284996012141	1.52923115989852
C	-0.00956157916877	2.64616849698140	1.87437353322755
H	-0.67119301718256	1.80480955540226	2.08133609378276
H	-0.44336515593925	3.21881368590699	1.05258083874703
H	0.02568652176423	3.29235017648722	2.74912042494211
C	2.49973529556418	-2.80324977197235	-2.03991654106967
H	2.38054507702945	-3.65875500738649	-2.68973194985027
C	3.75592702039667	-2.32089392290774	-1.71655810331783
H	4.64366227541902	-2.80206016579419	-2.10521207969560
C	3.87162643612606	-1.19959410769892	-0.90668172328296
C	2.71653786742939	-0.60712748400854	-0.42041001275765
H	4.84380631284608	-0.79446754723899	-0.67225914888440
Cu	-0.00000037551887	-0.00008867304261	-0.00000205394646

<u>OH</u>$^-$

55

OH

H	-4.94332811211974	0.31181996362058	-0.04963647905194
C	-3.42306291460840	-2.32122086031987	-1.51104297455425
H	-3.62616767954414	-3.26010269647100	-2.00686494859617
C	-4.45286132525656	-1.50998646410680	-1.06427573357826
H	-5.48452426291944	-1.80449974070368	-1.20632233403533
C	-4.15133446208705	-0.31879578605319	-0.42274350591117
C	-2.81792323694092	0.03616361970622	-0.24989257207387
N	-1.11580578288765	1.33647145425518	0.82940806774611
C	-0.68168672296456	2.32773263947149	1.61388524275622
C	0.75445994635428	2.31222033006646	2.04559082843501
H	0.85494095577549	2.74720418694574	3.03988525033101
H	1.14035090661267	1.29531770025238	2.05563568602099

H	1.37443586171134	2.90223753378876	1.36732103722337
N	-1.83027943408249	-0.76068817084868	-0.70006444421113
C	-2.10372359510722	-1.92154199078314	-1.31630014155593
C	-0.95531926203780	-2.76984775329661	-1.76509813028572
H	-0.43568195188363	-3.19445637859231	-0.90306143067703
H	-0.23668058653686	-2.16665697154138	-2.32082182021215
H	-1.30468602227182	-3.58873186221271	-2.39078567131128
C	-1.54946245332789	3.33696359344970	2.03644474496139
H	-1.18034843908807	4.13035180523814	2.67238865704839
C	-2.87411790795259	3.30211733223095	1.63598659794891
H	-3.56012555079833	4.08293970981967	1.93794479639885
C	-3.32428999964287	2.24106232806737	0.86184469434783
C	-2.41345175906810	1.25888933354743	0.48852963045436
H	-4.35990171427711	2.18883872783724	0.56242515057755
H	4.15271481801721	-2.42894055959182	0.76574838094679
C	1.18447347565150	-3.24121246432963	2.13984757614071
H	0.71238698187965	-3.95858415658485	2.79724596158926
C	2.52040312843998	-3.35498183533059	1.80187248281012
H	3.11384510992491	-4.18099233227899	2.17168840584671
C	3.10258874602524	-2.37691366867310	1.00831984062723
C	2.31113990850645	-1.33362378706794	0.54613263617230
N	1.97661121923658	0.72171343205559	-0.65165852448177
C	2.38643671529860	1.87309257454077	-1.20914630500354
C	1.36335244580007	2.87745442262877	-1.63385228840020
H	1.83957142034585	3.71671438269825	-2.13682819576279
H	0.63468932281747	2.39707831606038	-2.28593873746122
H	0.82233269889632	3.25734383643491	-0.76462843732085
N	0.98928585564314	-1.27703155691258	0.81402568553093
C	0.43159970459222	-2.18174322768203	1.63109135849817
C	-1.00678346097909	-2.02878266240449	2.01928218561455
H	-1.65861810826672	-2.58335577835363	1.34176499459434
H	-1.30142653072538	-0.98347688159080	2.00023419925608
H	-1.16109590398087	-2.42935310588660	3.02071247410913
C	3.74838416292203	2.12881196045020	-1.36482280093142
H	4.06256034751495	3.06030079097131	-1.81462993214282
C	4.67638825804199	1.19408011852000	-0.94031045697943
H	5.73614937429080	1.37713082446428	-1.06121843557437
C	4.23580997045276	0.02703899426053	-0.33563286790871
C	2.86884291661700	-0.17749947344841	-0.19307155811800
H	4.94688001499282	-0.69443446208800	0.03517526012618
Cu	0.06036958507035	0.01665543486108	-0.62281112418701
O	-0.09499733165829	0.39607086854015	-2.51078840021513
H	-1.00505549491810	0.23560710386925	-2.78205387907100

OOH⁻

56
OOH

H	2.91732066936633	0.52855311822117	-3.42948901824796
C	3.37822503689269	-1.76066694891268	-0.99529326335166
H	4.04915936635425	-2.54555533903497	-0.67494491863325
C	3.62391994552951	-1.03576641878007	-2.14994142797896
H	4.49586244868292	-1.24629787112835	-2.75556916145857
C	2.74117432439268	-0.03599015390190	-2.52730412847873
C	1.62845184076923	0.21633147991044	-1.73141223552550
N	-0.48802878307323	1.27535544232511	-1.31160062808978
C	-1.49733306013039	2.09991565771306	-1.60730676025727
C	-2.73048260853074	2.04285063609360	-0.75655686681130
H	-3.61939215432141	2.20719497951197	-1.36568690998214
H	-2.80716537241603	1.08027678212820	-0.25572570083982
H	-2.70873120216130	2.82338223918367	0.00716429601885
N	1.40509027677841	-0.50129706624798	-0.61841749923858
C	2.24774911715785	-1.46941461533042	-0.23633276506242
C	1.92588312207009	-2.22962111517630	1.01291808409658
H	1.08017388713141	-2.89939700294270	0.84186567165247
H	1.64954585311145	-1.54266196393490	1.81368143988083
H	2.77715358977993	-2.82896679571156	1.33012683046305
C	-1.40894927077465	2.98069497365606	-2.68685800103333
H	-2.23704052653026	3.63942030286402	-2.91139850871790
C	-0.25942477449677	2.99096490586205	-3.45731991456391
H	-0.16413579876246	3.67187809367332	-4.29326501222587
C	0.76757267370921	2.10456439251234	-3.16197506583810
C	0.61567394335197	1.24504823159759	-2.07960952326160
H	1.66045862551142	2.09155328628201	-3.76783187260022
H	-2.90550870484103	-3.16904611067504	2.97802792311822
C	-1.98953676797330	-3.73545003226953	-0.21531379067940
H	-2.01563407883347	-4.48485469665051	-0.99448524944925
C	-2.49084030530376	-3.99917304641994	1.04588418769959
H	-2.90149002917105	-4.97188550340059	1.28330921627901
C	-2.48796977259389	-2.99132287374942	1.99924527198918
C	-1.94964487904956	-1.75590731294821	1.66402805940533
N	-1.40213449463464	0.53545374637038	2.13790175215436
C	-1.51697954087405	1.68156416780342	2.82292043581866
C	-0.91011594300537	2.94105174064915	2.28709070684124
H	-1.58091209911911	3.77934683516166	2.47961292811844
H	0.03822575296895	3.14479309559970	2.78608682579790
H	-0.72028832132115	2.87251290704809	1.22039004656702
N	-1.39689051000608	-1.53226490655543	0.45334676891534
C	-1.44127973655491	-2.48194262535495	-0.49147483588407
C	-0.90734226025097	-2.18243410482079	-1.85781202194275

H	0.14462466768273	-2.46382524826733	-1.93364619158250
H	-0.99532953992205	-1.12396215854471	-2.08600111574073
H	-1.45625127071952	-2.75915786986121	-2.60153307723419
C	-2.21770037476901	1.71213487595007	4.02986890481437
H	-2.30445088646717	2.64776562212888	4.56521550712140
C	-2.79267470950301	0.55466213635776	4.51944736684193
H	-3.32959377543344	0.56142329302482	5.45899762094697
C	-2.69946260803670	-0.61433279116835	3.77743037397177
C	-2.00524683172696	-0.58989559527677	2.57574285712436
H	-3.17757638558943	-1.51720655280946	4.12390853083093
Cu	-0.10920009033908	0.16374713036215	0.59359346355060
O	1.26348357213616	1.12588340419092	1.60520655180200
O	2.07546703923018	1.98060152803118	0.77568658451866
H	1.57799603122860	2.81276380876122	0.78099848147071

O^{2-}

54
O

H	-4.93661887649907	0.42668024467504	0.18057792354664
C	-3.61369285170419	-2.30070715789331	-1.30172998808041
H	-3.87965627281425	-3.24999948371480	-1.74617679752973
C	-4.58541729877800	-1.44798081664281	-0.80364299790170
H	-5.63221225071138	-1.71998919217907	-0.84837197647092
C	-4.19977487568003	-0.24336550128197	-0.23673784586691
C	-2.84519152622154	0.07362344366930	-0.18483283848789
N	-1.11056839976898	1.31359825431298	0.91594346264998
C	-0.62590387772459	2.37727559693804	1.56198255527639
C	0.76857875310174	2.29119114682173	2.11094561672988
H	0.81482460253244	2.73266257471365	3.10740762793461
H	1.09646097747455	1.25560887691922	2.15616522002211
H	1.46705769329605	2.84224355859301	1.47691529725841
N	-1.91287343964194	-0.76132990877843	-0.67543792796035
C	-2.27266379719114	-1.93122154328566	-1.22668111103850
C	-1.18608293742313	-2.81155476117460	-1.75570668505317
H	-0.59183190117270	-3.22399913802292	-0.93697010117108
H	-0.52361094948385	-2.19698646087124	-2.37197069274259
H	-1.59877446919678	-3.63791158129496	-2.33237823672505
C	-1.39303433561156	3.53349093825984	1.72880980980922
H	-0.97892775602953	4.38352109152332	2.25517173132816
C	-2.67796673876125	3.57110965948033	1.21433394212367
H	-3.28513570149433	4.46156118351003	1.31628522949993
C	-3.18560308241093	2.44942056619387	0.57111185038270
C	-2.36908004090054	1.32880857286224	0.45078058161692
H	-4.18529138499331	2.45664125992484	0.16250117285421

H	4.27385147457924	-2.36250591923349	0.65781940266470
C	1.34969216733192	-3.49755391271714	1.90220952239311
H	0.91208980660954	-4.32714755169745	2.44160366035697
C	2.69098193981502	-3.49765645333617	1.56119224507393
H	3.32196930612084	-4.34146296074201	1.80938936686360
C	3.22549827347621	-2.39133171826639	0.91475165831495
C	2.38053697837109	-1.33242926373370	0.59919166097936
N	1.96078372399832	0.72637458824894	-0.57755583754451
C	2.31815181884427	1.91222051866803	-1.09434721652312
C	1.23862869992821	2.76118230919036	-1.68056181872094
H	1.65772563935138	3.59402045079742	-2.24370823420737
H	0.61854295621224	2.10992979300821	-2.31154875786751
H	0.60159532740339	3.16396353005315	-0.88868644512536
N	1.06430808241283	-1.36838788522989	0.87461875843468
C	0.55347833655619	-2.40580909728131	1.54740723533476
C	-0.89343601955371	-2.35810381709007	1.94151338047510
H	-1.50500974995923	-2.96083681443503	1.26684275981535
H	-1.26251247662473	-1.33662709930286	1.91446951925654
H	-1.02113479966074	-2.76525668796464	2.94526989419759
C	3.65130179925544	2.31900596095802	-1.07859037806193
H	3.91848344985970	3.28026586312243	-1.49589271014187
C	4.61253309443762	1.48896677800291	-0.52530612319691
H	5.65231631284004	1.78920915165481	-0.50292522203126
C	4.22603266596100	0.27168595012571	0.01455771956576
C	2.88001916839104	-0.08053670631461	-0.02222722841619
H	4.95434930385692	-0.37724082318507	0.47680792031936
Cu	0.04422237322920	-0.07857264221869	-0.79195478386268
O	-0.02868957481688	0.09860462329156	-2.63445119477965

H_2O

56
H2O

H	-3.28304807804998	2.27383813645258	3.52919845373387
C	-3.73145998110054	-1.03625974509087	3.05845162035894
H	-4.24930311629790	-1.89527444551282	3.46102264559149
C	-3.87229432339679	0.21647944526369	3.62943197090433
H	-4.51039068728139	0.35721535353039	4.49185311643948
C	-3.18661800647108	1.29372831186536	3.08957769256987
C	-2.37617992909558	1.08841386976649	1.98163440671506
N	-0.78893973911866	1.79219004380843	0.32713173852309
C	0.10679585384747	2.64395082671245	-0.19087294122178
C	1.01330097423358	2.16629247772766	-1.28216253847011
H	1.91558382096297	2.77498249321078	-1.30963433380649
H	1.28914854752140	1.12502215847927	-1.12780515485851

H	0.53070422132943	2.24973094108313	-2.25713704026841
N	-2.26195889575673	-0.13911266274462	1.43144122378702
C	-2.91176077328149	-1.19729387873114	1.94477961984604
C	-2.71357464052041	-2.54588913797386	1.32640677627941
H	-1.78247730941151	-2.99061280026638	1.68516555734118
H	-2.65746661160673	-2.49193032145598	0.24102514476563
H	-3.52915574474566	-3.20997261218252	1.60549393570181
C	0.20436454331803	3.94785471055043	0.29563766735113
H	0.93275237179483	4.62072597190213	-0.13521487939269
C	-0.62451865006118	4.35692422844100	1.32459329623767
H	-0.56935603194136	5.36789692041852	1.70642614109777
C	-1.50769006134867	3.44719110892745	1.88862915854626
C	-1.55476830465158	2.15857679098718	1.37511272208469
H	-2.12943368703323	3.73952437306048	2.72037296320101
H	1.61004643119450	-3.80072221214627	-2.08504793938708
C	1.66833636768698	-3.02384441179374	1.19515677203678
H	2.11852993533969	-3.31343878824005	2.13438818501349
C	1.92695875421599	-3.72847478520094	0.03347923589541
H	2.57159330122419	-4.59736715053168	0.04975596036388
C	1.37992447137632	-3.28944566324041	-1.16341167886068
C	0.55173081541287	-2.17597684529353	-1.14886868974590
N	-0.73728716609792	-0.44586683126353	-2.20914899185664
C	-1.19319627697631	0.24782092318386	-3.26251741703912
C	-2.00054297342332	1.49234859292283	-3.06044544646036
H	-1.59181703556048	2.29413755344454	-3.67726009877651
H	-3.02928159566334	1.32049117301017	-3.38013559397501
H	-2.02140603022243	1.81599371673514	-2.02546558528707
N	0.24802627021874	-1.54457251383296	0.00459792456011
C	0.82091911257119	-1.91684586415621	1.15831209975989
C	0.56406431906603	-1.11671948935430	2.39730293927439
H	-0.36216784211432	-1.42392831377635	2.88411622143129
H	0.49014935037539	-0.05626199966559	2.16425264745973
H	1.37482888097173	-1.26859733782535	3.10787991382674
C	-0.91036470241054	-0.18597752169023	-4.55812642834699
H	-1.27809612910740	0.39010298320560	-5.39601934135050
C	-0.17017911852438	-1.33534217719905	-4.75161963452923
H	0.04918521888372	-1.68628111199202	-5.75133306215419
C	0.30548250747953	-2.03239364774844	-3.64901945602899
C	0.01520957533650	-1.55892251238067	-2.37974794989693
H	0.90178575348354	-2.92086657423731	-3.78519587599126
Cu	-1.29142414428798	-0.17409208983528	-0.31149867728223
O	-3.35841934505737	-0.37758197763564	-1.16580460376879
H	-4.10795590631072	-0.30744553448147	-0.55948847552899
H	-3.50961448861757	-1.17977430291034	-1.68462328761298

tBuOH

68
tBuOH
H 3.92553291688007 2.82884034627811 -0.60301991129845
C 2.81206256977201 1.71581343653502 2.38022653474240
H 2.97247802652784 1.81423854786377 3.44456182260495
C 3.57243719064834 2.43218361209370 1.47354712844601
H 4.34197388760274 3.11032434302520 1.81771816907811
C 3.34102567519669 2.27538984509396 0.11501711945702
C 2.34458885601216 1.40421382619453 -0.29926822997733
N 1.02916560790185 0.27875081449036 -1.95544922224067
C 0.78925710615826 -0.17540392319667 -3.19417075379174
C -0.27235503131156 -1.21107467988061 -3.39583727115058
H -0.09851848125999 -1.74297030426552 -4.32946514506191
H -0.27568848357759 -1.92475160448874 -2.57363828825364
H -1.26120502752193 -0.75586150776039 -3.45378967942218
N 1.59894640871957 0.72984722117524 0.60282319429334
C 1.81482208712992 0.85841306480357 1.92145508131056
C 1.00068834475645 0.05325699823425 2.88436743290132
H 1.44618883680642 -0.93510164610371 3.01828074535468
H -0.02103503598691 -0.07923721258136 2.54043899665180
H 0.98375789853542 0.54188279927298 3.85656458921639
C 1.54267346032354 0.29088908177416 -4.27125559820269
H 1.33429819559829 -0.08356276497344 -5.26379283812738
C 2.55281017326066 1.21053770714248 -4.05356744684853
H 3.14234839132889 1.58487387175086 -4.88004131371271
C 2.83032476647359 1.62196694351054 -2.75762844671805
C 2.05510455018547 1.12339671488139 -1.72052347035065
H 3.64904481118348 2.29825548328856 -2.56675852402030
H -2.19395445957577 -4.36056172858270 1.41817066741974
C 1.16828288218060 -4.09986987339372 1.44839007571182
H 2.06548526118280 -4.64523573982543 1.70589359512233
C -0.08471781092204 -4.63493238847590 1.68827069735657
H -0.18684899673373 -5.60529277787295 2.15597339072978
C -1.21228321705471 -3.93223824401614 1.28862881597148
C -1.04607344964683 -2.68799958205459 0.69712021417215
N -1.83363563903234 -0.78440453315942 -0.54111265651729
C -2.77014147879441 -0.04723230801800 -1.15613673013845
C -2.38428242424657 1.16260603252495 -1.94580628506956
H -2.64206218426480 1.00983502001062 -2.99608957333976
H -2.95025893636165 2.02519481248892 -1.59759347921890
H -1.32771183898262 1.39436942385342 -1.87407614890062
N 0.17979328675995 -2.15038618611210 0.53130771078284
C 1.28167296990313 -2.84462917757126 0.85130695750706
C 2.62864920239751 -2.27229819440976 0.53586086327055

H	2.95425148709933	-1.57044115431938	1.30452138706731
H	2.61372975923352	-1.74679191059771	-0.41705238791870
H	3.36520836345432	-3.07225204380384	0.48746089333992
C	-4.11310032558277	-0.41636470814394	-1.07694521978715
H	-4.85364100166450	0.18921755968967	-1.58096056369144
C	-4.47671768858083	-1.53687402760378	-0.35632861872466
H	-5.51641522519165	-1.82515651349445	-0.27511714098041
C	-3.49233465971016	-2.29852442758718	0.25915818640684
C	-2.16919583146046	-1.90612327237530	0.14142247357794
H	-3.75873277233856	-3.18052808044542	0.82002028632860
Cu	0.03832144124957	-0.20688225131766	-0.17965246418045
O	-1.25109452977987	1.54021662078283	0.97552563610987
C	-1.32178222894237	2.96119571121981	1.30723078402704
C	-2.76379368545947	3.42739338366443	1.12399700598284
C	-0.38840048864467	3.66703542754160	0.33630967625642
C	-0.87596209059837	3.16060080517489	2.75282013685307
H	-3.43663227617509	2.84766468730176	1.76066899172909
H	0.15882709380617	2.84914298398972	2.88828673754455
H	-0.65501849424846	3.43269848269673	-0.69439885422583
H	-2.85757709868056	4.47897127548716	1.39993257988711
H	-0.46159924478064	4.74619641094303	0.47546782983966
H	-0.95486073374210	4.21366614133588	3.02851185343396
H	-3.08035892392300	3.31449828008366	0.08771963684394
H	-1.50755564551224	2.58231465472974	3.43084085804342
H	0.64636347836882	3.37467207592966	0.50662311386003
H	-1.89725179604813	1.07600827317389	1.52380609733755

tBuO⁻

67
tBuO

H	3.92542061377880	0.31420031221015	2.79022587472196
C	0.80052521333444	0.19391559034636	4.05591163580888
H	0.25359066083286	0.18880022855426	4.98835736389107
C	2.18274056967715	0.26896571501339	4.03800535178426
H	2.74190424410451	0.31873574649838	4.96319894625716
C	2.84757702535879	0.27500813296528	2.82126337870532
C	2.10544279514712	0.20402430105268	1.64816408411265
N	1.95808513510336	-0.23760554413679	-0.70670878479174
C	2.47012000404640	-0.39590005382697	-1.92961775936259
C	1.58197157944327	-0.93505580466384	-3.00994706588398
H	2.00577261633805	-1.85874161904827	-3.41035436244027
H	0.58734933952144	-1.14315540910004	-2.62535495026231

H	1.50314491634728	-0.22939338807096	-3.83851651815741
N	0.76263546047372	0.13830833190242	1.68546516304816
C	0.10264593432367	0.13357641246452	2.85262626779348
C	-1.39145893686981	0.05951887738025	2.82577287481621
H	-1.71994343947855	-0.96918837622473	2.66000865648058
H	-1.78584083868296	0.67623272374989	2.01891109743709
H	-1.80638449645391	0.39503965104352	3.77456268885706
C	3.81112711768010	-0.09984311522983	-2.18997027870683
H	4.20287045502402	-0.22638471501814	-3.19051258410073
C	4.61775055478854	0.34733902045276	-1.15954061193896
H	5.65542560572646	0.59549664470179	-1.34123804658029
C	4.08641578358418	0.46289492854200	0.11919306709965
C	2.74643579162172	0.15141432671663	0.31138810053092
H	4.70427400539980	0.80261249755748	0.93633911596914
H	-4.14948341056156	-2.88125003532592	-0.14212716580404
C	-1.33706395743877	-3.87612329198848	1.42543762923511
H	-0.94523228291792	-4.64664323113657	2.07549349469520
C	-2.67265789072057	-3.85514581872832	1.06957854920575
H	-3.35795892268047	-4.59800168662487	1.45646421160036
C	-3.11970014766255	-2.88843733738653	0.18013224456168
C	-2.21560224370490	-1.94515097574577	-0.29003792643244
N	-1.60660370089545	-0.05724132794696	-1.64045290396957
C	-1.77298261386111	0.79030428464614	-2.66252169897846
C	-0.66232182305762	1.71038197847876	-3.05458528280546
H	-0.41572744529724	1.55404545567472	-4.10708646269639
H	-0.97277100012336	2.74891789229603	-2.93848397487888
H	0.21997873583167	1.55037949275716	-2.44838750771199
N	-0.93341773365755	-1.92223384522098	0.13052752071571
C	-0.47704512966639	-2.89153169320806	0.93578557306921
C	0.97844008557171	-2.93084059264020	1.28660599191687
H	1.15838057740567	-2.49701706529992	2.27207037034363
H	1.56785268056383	-2.38887519666854	0.55365408112582
H	1.31570499143377	-3.96694062284320	1.32336656721229
C	-2.96650105735704	0.79730886100920	-3.38792903952141
H	-3.07899666913950	1.48679731731339	-4.21377784106351
C	-3.98084276066969	-0.07580506765350	-3.04560538479864
H	-4.91587062198198	-0.07519721234089	-3.59052406614641
C	-3.77854333839622	-0.97590966036051	-2.00807430082627
C	-2.56759062670650	-0.95174304726358	-1.33020842318188
H	-4.54495543379369	-1.69082104717350	-1.75259305429175
Cu	-0.22749893641206	0.13148728240761	-0.09854103109656
O	-0.76954923207298	1.97456271580535	0.01459347712763
C	-0.12260483625475	3.17765526949375	0.34734598457260
C	-1.07111231456442	4.30365718694419	-0.10367957389218
C	1.21980421667570	3.33751281312344	-0.38257380708292
C	0.11074646686609	3.32579647863300	1.86155544278355

H	-2.02428873029727	4.22186942797531	0.42361983913835
H	0.88174866079404	2.64415389789161	2.21600950883111
H	1.07687524397357	3.33036353573785	-1.46284499038755
H	-0.64381993615688	5.28793380561241	0.10433692511117
H	1.70170276751173	4.27917005980131	-0.10789347498811
H	0.43115277713149	4.34220708967741	2.10396858993693
H	-1.26328794469916	4.22740734306020	-1.17497912946922
H	-0.81280453106212	3.11892998684278	2.40592948551927
H	1.89846817308003	2.52671359674249	-0.12247384316709

tBuOO⁻

68

tBuOO

H	-3.89701530714462	-2.44016060370845	0.79400413766098
C	-1.98497490870701	-2.99758118253382	-1.92984231153066
H	-1.93639621598293	-3.58317700396142	-2.83730674635016
C	-3.04658148052208	-3.12685157797208	-1.04987273033443
H	-3.84546114244688	-3.82790079819245	-1.25357038427774
C	-3.08060516637455	-2.34524894034677	0.09477595532783
C	-2.03730907177474	-1.45826009692728	0.33580609359121
N	-0.78060510225724	-0.07337421551022	1.83753513833530
C	-0.60170171894527	0.62323637861709	2.96308771832403
C	0.76898396480210	1.15883763789408	3.24996210978206
H	1.01374444582040	1.03190871780755	4.30513278244601
H	1.51389449703631	0.64999650362556	2.64218558873968
H	0.81917225821322	2.22718094575157	3.02952730559087
N	-1.00757493822002	-1.35999484793445	-0.52185754343934
C	-0.96747505439095	-2.09127257293173	-1.64251505773974
C	0.19852851487321	-1.90692147913322	-2.56289074674345
H	1.08493740925241	-2.40732490089831	-2.16666934054547
H	0.43321380368793	-0.84729402629071	-2.67051276907099
H	-0.01745674868521	-2.32823078973753	-3.54301620817171
C	-1.66030108560092	0.83580274453749	3.84847348899696
H	-1.49638230527663	1.40320193487254	4.75489719104450
C	-2.90696925231829	0.31390624581190	3.54855835384834
H	-3.74570639524229	0.48049690449720	4.21214368200247
C	-3.07131504288616	-0.43947683194754	2.39339477498775
C	-1.97478552648028	-0.62352629212417	1.56010551323224
H	-4.03580791423601	-0.85870416098396	2.15000031204649
H	5.11631601150884	-0.97108148067822	-1.10183110753677
C	3.12918464627157	-3.34860475995760	0.22554533027319
H	3.10142432479079	-4.40010772578745	0.47702015410786
C	4.23155940246273	-2.79260646498950	-0.39672877043394
H	5.08108280334512	-3.40727398210324	-0.66446743057551

C	4.25051940080575	-1.42785557309321	-0.64783077560800
C	3.14271538323637	-0.66879535587339	-0.29561296634271
N	1.94801848250736	1.40364137005140	-0.08712915963555
C	1.86113810890217	2.73872971013357	-0.01993930023497
C	0.59267930297346	3.39342003339315	0.43216373161003
H	0.82327995096930	4.10470523852075	1.22818904790860
H	0.14324601516229	3.95136299403618	-0.39065938281175
H	-0.13721897470556	2.67353030120884	0.77958533493597
N	2.03979039910060	-1.23577276218923	0.23751211746828
C	2.03292632485768	-2.54243047759160	0.53644043548104
C	0.84137945590738	-3.13057124213605	1.22677212576116
H	0.16605141072705	-3.59755724731650	0.50707676528699
H	0.29298400317546	-2.36764302779718	1.77107854213321
H	1.16568164900834	-3.90649358553857	1.92008726633312
C	2.96649244410339	3.53167820033481	-0.33922672310313
H	2.87822396619951	4.60785864782310	-0.27590884368511
C	4.15338160526864	2.93628381480509	-0.71953881946727
H	5.01492435941476	3.53905900476621	-0.97569323527951
C	4.23921265182175	1.55056868774977	-0.74209674849723
C	3.11653849826298	0.80723585967224	-0.40820178736196
H	5.17061261509126	1.06807092447594	-0.99469809480670
Cu	0.38251540480458	0.07248216649380	-0.08585913206726
O	-0.60792136250071	1.29944210058114	-1.22225358512188
O	-1.91248734569937	1.51557312291399	-0.63863287899246
C	-2.90141620065415	1.73760072021830	-1.66415170061325
C	-4.18014424090499	1.93811984546266	-0.85465130510438
C	-3.02687034936867	0.52549357371195	-2.58219282793958
C	-2.55213770144491	2.99618580988334	-2.45774690307308
H	-2.43782541007068	3.84855227687887	-1.78504268354228
H	-4.40149014203216	1.04770590471097	-0.26391428878067
H	-2.07121029187215	0.30737856620478	-3.05518734110698
H	-1.61920630524672	2.85598008451244	-3.00314593103769
H	-4.07919128334128	2.78998148949456	-0.17971471598557
H	-3.34893481603298	-0.35038389274579	-2.02011001864494
H	-3.34252415347466	3.22332848799430	-3.17577288579260
H	-5.01894921389343	2.12586053003882	-1.52667013801312
H	-3.76429334563021	0.72334541944569	-3.36248967785663

H^-

54
H

H	4.39199274535072	0.21102180012441	-2.35652120532633
C	4.22590777643272	0.82236850333674	0.95483304795500
H	4.84223533409331	1.03819795863574	1.81646205314215

C	4.78635736653813	0.65085875552910	-0.30046928755904
H	5.85618120170079	0.73423175236196	-0.44084695513976
C	3.96402323796030	0.36195215856904	-1.37792498514873
C	2.59172645551250	0.25652530479001	-1.17172736582476
N	0.36596294758513	-0.29038814868652	-1.89785967370500
C	-0.53020997979277	-0.72670772312283	-2.79159016547917
C	-1.93186941513753	-0.97595157621400	-2.32408845439545
H	-2.44109197524984	-1.66563973114347	-2.99561868621484
H	-1.93097308751805	-1.38504624403685	-1.31556636806038
H	-2.50285620751795	-0.04565970011939	-2.30467920084142
N	2.06337637377639	0.44144198680013	0.05219402469060
C	2.84661958244571	0.71337144330528	1.10751335817835
C	2.19106315896875	0.88677712838308	2.44031767524247
H	1.66020594033401	-0.02282321348980	2.72833081401087
H	1.45155996366755	1.68876391638242	2.39033264998948
H	2.92717760187302	1.12051231682278	3.20697656393513
C	-0.16739830575062	-0.95294971294804	-4.11955635380829
H	-0.90741671052645	-1.30882240609191	-4.82340337948044
C	1.13981930472833	-0.72257873204086	-4.51269245636747
H	1.44178178533097	-0.88229570169680	-5.53972601648979
C	2.06831258678384	-0.30395013563979	-3.57126959311161
C	1.64892427235822	-0.10872195194647	-2.25842619459476
H	3.09484127957410	-0.14404309180680	-3.86259644783889
H	-2.94703962026524	-1.69240545499901	3.51565596822908
C	-0.10531224590310	-3.21269391662822	2.53113481846111
H	0.52441410154431	-4.06784565581668	2.73559416349380
C	-1.24042199224390	-2.96537935019838	3.28226886575487
H	-1.50910775413697	-3.61557646155992	4.10468974303683
C	-2.04639531897171	-1.88574173335489	2.95367255512917
C	-1.66985369728249	-1.06784288303279	1.89440718791795
N	-1.99831739335686	0.83090451049261	0.45332589198147
C	-2.72786946245281	1.81151379790901	-0.10071115242804
C	-2.11668404067651	2.65043819087021	-1.17825635286290
H	-2.89477062922465	3.12992094727812	-1.77070019999416
H	-1.48841981877320	3.42684755542043	-0.73520493634133
H	-1.47879850135784	2.05660697484635	-1.82978000577167
N	-0.52795287242449	-1.27831327324625	1.21046436666974
C	0.23296493736087	-2.34525202775285	1.49159770732866
C	1.45138482027846	-2.60216925139962	0.65942601498493
H	2.31060371650934	-2.05384610799312	1.04994564434818
H	1.28863226486098	-2.28594195218802	-0.36799166540326
H	1.69923975200319	-3.66261149338140	0.67604285315719
C	-4.03255173862945	2.04953041298182	0.32750679276760
H	-4.60730593738528	2.83827168051550	-0.13799067374165
C	-4.57325855190552	1.27222377244274	1.33691574029552
H	-5.58344462365995	1.44612797945151	1.68391722446675

C	-3.81290282512564	0.25191516466450	1.88716734174781
C	-2.52021127402151	0.04795281874757	1.41776416832515
H	-4.23223109881076	-0.38244707927218	2.65230871564297
Cu	0.01092659504702	0.56159213849815	0.14145700565416
H	0.22220943208245	2.11289701644767	0.36294273729216

Cl^-

54
Cl

H	-4.49924077475653	-4.18060684694594	3.63416449776430
C	-4.51513230694694	-2.73678690627011	0.58612770075214
H	-5.15150604507652	-2.54242675322181	-0.26583579377717
C	-4.99398744532488	-3.40665445461069	1.69870996674368
H	-6.02105246425284	-3.74547112473900	1.73526302901310
C	-4.14377056445665	-3.64659344614542	2.76705772346159
C	-2.83027790530693	-3.19980609683833	2.69592947235579
N	-0.56655310465569	-3.11441844930686	3.48065450855706
C	0.42781712950186	-3.44187674166270	4.31385599014192
C	1.83384265059916	-3.11203946742974	3.91549279015969
H	2.51964296665916	-3.86208873412802	4.30843846571850
H	1.92900966182246	-3.06845418585794	2.83294570204651
H	2.13797129959803	-2.14660537459350	4.32395482366762
N	-2.38952592593603	-2.53371274695822	1.61120041775266
C	-3.19334780810102	-2.29978106598129	0.56331482974072
C	-2.63624227888309	-1.58735803775127	-0.62863932613874
H	-2.06014293239191	-2.27831240001040	-1.24873753462341
H	-1.97683857825580	-0.77656958541497	-0.32335774363085
H	-3.44174652435741	-1.18281380899994	-1.23879545970399
C	0.16009759884382	-4.09274429529494	5.51909651830922
H	0.97682114381065	-4.35008922347560	6.17973879055047
C	-1.14761430826300	-4.40337790483400	5.84742914824564
H	-1.37720912075421	-4.89421819432755	6.78422462323357
C	-2.16460818628706	-4.10373035826450	4.95127202756220
C	-1.83526840766698	-3.46912204315753	3.76005837611108
H	-3.18505510689483	-4.36800889182701	5.18205294713904
H	2.68085114776468	-1.26722579223220	-1.89182760062301
C	0.76971054358773	-4.03163304605460	-1.62972409701427
H	0.53341071449867	-4.95890064876029	-2.13324328676319
C	1.63978789699177	-3.11595723374455	-2.19262891219467
H	2.08398679989264	-3.30314477679075	-3.16150592940795
C	1.96804223839854	-1.96800169058298	-1.48579778687595
C	1.37823505056973	-1.75680452515463	-0.24694632975802
N	1.13077497552253	-0.59054166370889	1.83984187921480
C	1.51101340630841	0.28902815092132	2.77688923602148

C	0.86172915057301	0.28987537821826	4.12569624814661
H	1.63441348887194	0.27071886635803	4.89670253056403
H	0.27985646049911	1.20234668010815	4.25795591955800
H	0.19477486622256	-0.55387439271381	4.26359978725124
N	0.47133092535169	-2.62035900370178	0.25552641310227
C	0.19253166626547	-3.76207403986948	-0.38820827458337
C	-0.72562290266461	-4.75890584489313	0.24825451040066
H	-1.76138701426077	-4.58475331873182	-0.04828607941040
H	-0.66635031431255	-4.70457426067637	1.33192416907981
H	-0.45633181115131	-5.76327666799408	-0.07614984231597
C	2.53252803215243	1.20165348819177	2.50894508327414
H	2.82911324537802	1.89855827979899	3.28083154226525
C	3.14370079876104	1.20537203803056	1.27043554205796
H	3.92459128089390	1.91923183190047	1.04345597752306
C	2.76263215187187	0.26379009445280	0.32454859763255
C	1.76081941573539	-0.63989872109415	0.64353994493331
H	3.25399035620999	0.22921188044453	-0.63499162707721
Cu	-0.58242153166410	-1.66617477838865	1.82799991824232
Cl	-1.81896902738283	0.31829727351118	2.27157446908265

CH_2Cl^-

57
CH2Cl

H	3.63315580738970	3.86413692761397	-2.45359766787503
C	3.78105209564603	2.89036654267339	0.77192204777371
H	4.33620782624829	3.04145964486530	1.68752153221912
C	4.10714937497374	3.57868108243194	-0.38472051578502
H	4.92681989355133	4.28533227094025	-0.39070249981164
C	3.37742942984931	3.34877704638191	-1.54081354396205
C	2.32575613453025	2.43959179471304	-1.50515768843540
N	0.68038159086912	1.06472025984823	-2.58855478676552
C	-0.01144524194933	0.63299915905134	-3.64797331618361
C	-0.87180925981522	-0.58424601986965	-3.48728602756102
H	-0.74057454039951	-1.25263226101577	-4.33933080370404
H	-0.62182004369928	-1.11487101394126	-2.57217492017018
H	-1.92798817813443	-0.30964492227847	-3.45060481884878
N	2.01883439213632	1.78476082971327	-0.37061856346199
C	2.72404472148362	1.98336701697609	0.75127288122845
C	2.37681891316584	1.17798160377950	1.96531876231326
H	2.89328279169891	0.21512982098117	1.93363731184764
H	1.30854049883429	0.97485423830895	2.01583777503206
H	2.68945112198400	1.69392748592723	2.87177157599275
C	0.07829237884887	1.28643523355284	-4.87850908489718
H	-0.49138798464234	0.92070963389114	-5.72238090980268

C	0.90258439546309	2.39145018881776	-4.99823754222321
H	0.97851075437554	2.92120087164452	-5.93903559994970
C	1.64853674835393	2.80567334130174	-3.90311338130651
C	1.52145965263748	2.10825072316355	-2.70720073249823
H	2.30699552505016	3.65649550391217	-3.98791495850136
H	-0.01642374718129	-3.39050445026708	2.29789204986316
C	2.83887733916682	-2.66786884681152	0.65522489898590
H	3.87404676524719	-2.96802650155798	0.56345676106498
C	1.99716954831944	-3.27966119185356	1.56618391123725
H	2.36722489048038	-4.05691052595900	2.22209025026660
C	0.66133348632880	-2.90466598078573	1.61263103940276
C	0.22234941862722	-1.90006299664722	0.75976787307331
N	-1.47142320446627	-0.41495266829082	-0.08701525208418
C	-2.73755918692527	-0.07521945495167	-0.36302867294794
C	-3.01373975642396	1.11325014563130	-1.23177734414834
H	-3.75307039797832	0.84890179025308	-1.98904001430037
H	-3.43184688101837	1.93074674466759	-0.64102432697940
H	-2.11537847351898	1.47296857832927	-1.72165520906787
N	1.06846602168286	-1.25330456131848	-0.06646654546973
C	2.34577836942840	-1.64422820581009	-0.15651410471444
C	3.23813758892173	-0.97627547006537	-1.15740512731196
H	3.85915886275835	-0.21668103464515	-0.67861831598897
H	2.65124525354229	-0.49928597012181	-1.93689362076067
H	3.90803302828549	-1.71128408345654	-1.60453027946680
C	-3.79995010722476	-0.82635136867957	0.14191085934584
H	-4.81355018979301	-0.53578513696028	-0.09903408115163
C	-3.54179526915488	-1.92944087568625	0.93336745264664
H	-4.35381263685267	-2.51950777671756	1.33778068551086
C	-2.22477222398168	-2.29089623965815	1.18144926574099
C	-1.20432423733812	-1.51751306378914	0.64495417395604
H	-2.00426933828624	-3.17569924850953	1.75853349186031
Cu	0.22074592474493	0.74322825020972	-0.41350000682654
C	-0.61358521972928	2.39882335655415	0.46007107808637
Cl	-0.67574731754898	3.83230648338437	-0.65973101454905
H	-0.00233257973787	2.71506056982116	1.30109072064801
H	-1.63525698112372	2.22670956440770	0.78413245161510

$CHCl_2^-$

57
CHCl2

H	1.57952830960395	4.23531203487279	-1.72991238666965
C	3.36894557353037	2.55686180230636	0.58154048899089
H	4.28500529830780	2.60158535635812	1.15425952347417
C	3.01657043470525	3.57585627295479	-0.28504435008660

H	3.65171791258514	4.44461383431839	-0.39952823660174
C	1.84799044039781	3.46547578028387	-1.02372528706344
C	1.04441496662860	2.34512635492215	-0.85182740722334
N	-0.73963654219371	0.90004310655916	-1.55957824114110
C	-1.77892278033644	0.56794867848743	-2.33138014714084
C	-2.30250891153075	-0.83507195033879	-2.25571609584871
H	-2.34232125781083	-1.27188211615045	-3.25533323752380
H	-1.66726049241253	-1.45102329068794	-1.62510776828243
H	-3.31774415279314	-0.85058183037237	-1.85536636534447
N	1.38724927570500	1.37806626902652	0.02219641219330
C	2.53416654773368	1.44846091298601	0.71229555478684
C	2.94436177283693	0.29115561742591	1.57015326113542
H	3.54389355570553	-0.40537132036365	0.97806874999292
H	2.08799953511468	-0.24971278357733	1.96240159714556
H	3.55670084765729	0.63397850066434	2.40255758960203
C	-2.34537989551057	1.49532884620350	-3.20804919494309
H	-3.18852336099067	1.20788636168402	-3.82205001813547
C	-1.81142630502044	2.76966677847540	-3.28036902979462
H	-2.24197889077621	3.50921418685023	-3.94309654637368
C	-0.70355398271557	3.08958402457552	-2.50688268112236
C	-0.18140653265750	2.12047336901716	-1.65840784725908
H	-0.27053029798337	4.07627516959897	-2.56564911332536
H	1.46140243264241	-4.56713341044516	1.51585566966333
C	3.02723520773972	-2.83675094677369	-0.91653339736560
H	3.89215598885247	-2.86379870083609	-1.56527764133159
C	2.81443531750808	-3.82263281163545	0.02884252652326
H	3.52419201673940	-4.63024139609828	0.15209477738060
C	1.66085978449868	-3.78471844254879	0.80005609451886
C	0.77283785296854	-2.73307870444148	0.61810309828557
N	-1.29342168727523	-1.57607149540476	1.03238689077724
C	-2.57308427885758	-1.52093731255527	1.42617493996825
C	-3.42969878135496	-0.35736469756849	1.02673081280056
H	-4.30524661512765	-0.72746256775242	0.48995153663713
H	-3.79422675484807	0.17721339626031	1.90593835370019
H	-2.90235799546335	0.34099141447652	0.38547376681915
N	1.03176273109017	-1.73187302424173	-0.24854020476243
C	2.11351711709252	-1.78785997328287	-1.03632439408498
C	2.32673817708500	-0.72019379791100	-2.06463139616868
H	3.02462199476975	0.03648696957018	-1.70085503325122
H	1.39013231424083	-0.23184081360702	-2.31626671391636
H	2.76121871510405	-1.15883843515913	-2.96307671885977
C	-3.12959115890459	-2.55870054504441	2.17544969075675
H	-4.16416724758444	-2.49149803614629	2.48354268170240
C	-2.35602480459901	-3.65482507658505	2.50533360765783
H	-2.76751799431835	-4.46195559579014	3.09710019956816
C	-1.05128333639980	-3.72786337305120	2.03668874747503

C	-0.54963371174700	-2.67689142783904	1.28267117076045
H	-0.45104016174579	-4.60119652894108	2.23885619761628
Cu	-0.09314644696287	-0.00717636785912	0.39615995046093
C	-0.82368481897108	1.21874796530693	1.90562167247124
Cl	-1.48703247316520	2.79052058874278	1.29837567435105
Cl	0.35150511157837	1.56298037295843	3.23848505041655
H	-1.65544441636537	0.75221898312270	2.41683225388850

CH_3CN

59
ACN

H	-4.51677127055667	-4.02901025283616	3.76118392726819
C	-4.58208900800630	-2.60109085518994	0.70560654721676
H	-5.24486850984217	-2.37039843588098	-0.11659341067728
C	-5.04770770422340	-3.23768192393928	1.84314331450986
H	-6.09131377401487	-3.51006995565717	1.93035788772796
C	-4.16573630044848	-3.52835105149122	2.87256841740483
C	-2.83217518140249	-3.16394096577943	2.73921764888296
N	-0.55133969104061	-3.06139646856810	3.48394294475395
C	0.46900726802301	-3.42089909949167	4.27499177267855
C	1.85870000143996	-3.00799602686549	3.90089839422216
H	2.58230045108971	-3.68850838053025	4.34699115365481
H	1.98730502124203	-3.00761655215469	2.82061834693074
H	2.07988742478783	-2.00522000471705	4.27051179897891
N	-2.40424235140737	-2.52455755252308	1.63177751922977
C	-3.23928311761206	-2.24421926025735	0.61899554825620
C	-2.69629843257647	-1.57661796729940	-0.60558393984211
H	-2.20278033792721	-2.31122716936777	-1.24662184983717
H	-1.96527253876442	-0.80984999671302	-0.35656102477705
H	-3.50349456667711	-1.12298780576351	-1.17736715759655
C	0.24271960439167	-4.18260669470852	5.42205853427010
H	1.08009291233008	-4.46295375661366	6.04606508226806
C	-1.04481767683184	-4.57596344084159	5.73820908162477
H	-1.23861579405206	-5.16166039675838	6.62731980154095
C	-2.08612800302311	-4.23705602417541	4.88616825407989
C	-1.80351440852621	-3.48481490439552	3.75372742030665
H	-3.08982426125307	-4.57001788772132	5.09905171768638
H	2.88796376600986	-1.40465529761772	-1.76999118099996
C	0.85580008406050	-4.08900312358707	-1.60955338019144
H	0.62150760769054	-5.01553513407621	-2.11506651826506
C	1.79892935905136	-3.21682487415922	-2.12121520672199
H	2.30563926469780	-3.43827959003105	-3.05135372444018
C	2.11889011851827	-2.06986855188410	-1.40942642912111
C	1.44702841555590	-1.81221449830630	-0.22294367702513

N	1.10257339643674	-0.60294738304564	1.82346457012817
C	1.41028515975006	0.31682225281790	2.74904084049837
C	0.65734550590633	0.37116875127659	4.04251813784166
H	1.36524543971616	0.37535388541507	4.87291026261452
H	0.08082033934300	1.29609838013580	4.10125446094221
H	-0.02381742628406	-0.46430895717933	4.16664300651207
N	0.47464236026806	-2.63455670815948	0.22617303984445
C	0.20205905453134	-3.77950907943134	-0.41654049356909
C	-0.78536664417550	-4.73874030368279	0.17190250044312
H	-1.80548457403994	-4.49434815143783	-0.12781058941772
H	-0.73719496080598	-4.72660620181297	1.25821656642938
H	-0.57193216591178	-5.74604686848252	-0.18208114053141
C	2.44477153760721	1.22343330894021	2.51680620307474
H	2.68452151082865	1.95292292552015	3.27824764722575
C	3.14169982085452	1.17833561702239	1.32511409662485
H	3.93844932362393	1.88306432767785	1.12672343269316
C	2.82128443364670	0.20714032284401	0.38638573540229
C	1.79845547461671	-0.68559160839584	0.66614531019999
H	3.37119424837049	0.14572131029710	-0.53939686652599
Cu	-0.56761225542965	-1.72198414229575	1.79897333260357
N	-1.64861327640386	0.08598864067444	2.04821082644710
C	-2.27176758372291	1.03831990608093	2.17990912811894
C	-3.05162970719742	2.24291386737199	2.35168611215877
H	-2.43043622035710	3.01477640383946	2.80807700257740
H	-3.40633069434987	2.58874277012979	1.37989608737091
H	-3.90563600612442	2.03412930338155	2.99729646369521

Appendix III: ORCA input examples

g-tensor:

```
!PBE0-DH  ZORA  DefGrid3  UKS  ZORA-def2-TZVP  Def2/J  NoFrozenCore
KeepDens

%cpcm
  epsilon 22.84
  refrac 1.384
end

%rel
  SOCType 3
  SOCFlags 1,3,3,0
end

%basis
  NewGTO Cu # new basis for copper
    "ZORA-def2-TZVPP"
  end
end

%basis
  NewAuxCGTO H
    "ZORA-def2-TZVP"
  end
end

%basis
  NewAuxCGTO C
    "ZORA-def2-TZVP"
  end
end

%basis
  NewAuxCGTO N
    "ZORA-def2-TZVP"
  end
end

%basis
  NewAuxCGTO O
    "ZORA-def2-TZVP"
```

```
  end
end

%basis
  NewAuxCGTO Cu
    "ZORA-def2-TZVPP"
  end
end

%eprnmr
  gtensor true
end

#insert here geometry input
```

A-tensor:

```
!B3PW91 D3BJ UKS def2-TZVP AUTOAUX NoRI

%cpcm
  epsilon 22.84
  refrac 1.384
end

%rel
  SOCType 3
  SOCFlags 1,2,3,1
end

%method
  SpecialGridAtoms 29
  SpecialGridIntAcc 11
end

%basis
  NewGTO Cu
    "aug-cc-pVTZ-J"
  end
  Decontract true
end

%eprnmr
   Nuclei = all Cu {Aiso, Adip, Aorb}
end

#insert here geometry input
```
